# Refining penalized Ridge regression: a novel method for optimizing the regularization parameter in genomic prediction

**DOI:** 10.1093/g3journal/jkae246

**Published:** 2024-11-09

**Authors:** Abelardo Montesinos-López, Osval A Montesinos-López, Federico Lecumberry, María I Fariello, José C Montesinos-López, José Crossa

**Affiliations:** Centro Universitario de Ciencias Exactas e Ingenierías (CUCEI), Universidad de Guadalajara, Guadalajara 44430, Jalisco, México; Facultad de Telemática, Universidad de Colima, Colima, Colima 28040, México; Facultad de Ingeniería, Universidad de la República, Montevideo 11300, Uruguay; Facultad de Ingeniería, Universidad de la República, Montevideo 11300, Uruguay; Department of Public Health Sciences, University of California Davis, Davis, CA 95616, USA; AgCenter, Louisiana State University, Baton Rouge, LA 70803, USA; Department of Statistics and Operations Research and Distinguish Scientist Fellowship Program, King Saud University, Riyah 11451, Saudi Arabia; Colegio de Postgraduados, Montecillos, Edo. de México CP 56230, México; International Maize and Wheat Improvement Center (CIMMYT), Texcoco, Km 45, Carretera Mexico-Veracruz, Edo. de México CP 52640, México

**Keywords:** ridge regression, genomic prediction, GenPred, Shared Data Resource, plant breeding, breeding values, penalized regression

## Abstract

The popularity of genomic selection as an efficient and cost-effective approach to estimate breeding values continues to increase, due in part to the significant saving in genotyping. Ridge regression is one of the most popular methods used for genomic prediction; however, its efficiency (in terms of prediction performance) depends on the appropriate tunning of the penalization parameter. In this paper we propose a novel, more efficient method to select the optimal penalization parameter for Ridge regression. We compared the proposed method with the conventional method to select the penalization parameter in 14 real data sets and we found that in 13 of these, the proposed method outperformed the conventional method and across data sets the gains in prediction accuracy in terms of Pearson's correlation was of 56.15%, with not-gains observed in terms of normalized mean square error. Finally, our results show evidence of the potential of the proposed method, and we encourage its adoption to improve the selection of candidate lines in the context of plant breeding.

## Introduction

The popularity of genomic selection (GS) continues to grow in plant and animal breeding due to the introduction of large-scale molecular genetic data that is used to train statistical machine learning models that are used to predict complex traits for which only molecular data is available. GS has proven to be successful for traits on many species (maize, wheat, groundnut, cotton, rice, soybean, etc.) in plant breeding, along with many successful applications in animal science. However, the GS is still not optimal for many plant breeding programs since many factors affect its accuracy. Some of these factors are the degree of relatedness between training and testing, quality and coverage of the markers, population size and architecture, the heritability of the target trait, prediction model, goal of prediction (tested lines in tested environments, untested lines in tested environments, untested lines in tested environments and untested lines in untested environments), genetic architecture, etc.

For this reason, research continues to optimize the GS methodology since its practical implementation requires good prediction accuracy. From the modeling point of view, many statistical and machine learning methods (linear models, mixed models, random forest, support vector machine, Bayesian methods, deep learning, etc.) had been explored for genomic prediction (Montesinos-López *et al*. 2022), but linear and mixed models are still among the most popular due to their robustness, simplicity, ease of implementation, and interpretability, as well as the fact that these models make it easier to account for genotype-by-environment interactions and integrate multiomics data.

Regarding many machine learning models used in genomic prediction, in many cases, they produce similar or better results than linear or mixed models, but at the cost of a significant effort in the selection of the optimal hyperparameters; for this main reason, these models are still less popular in plant and animal breeding than linear and mixed models (Montesinos-López *et al*. 2021; de los campos and Gianola 2023). For example, deep learning models are the most difficult to train efficiently since they generally require a large sample size and many computational resources for a successful implementation, since this model has a lot of hyperparameters to be tuned (Montesinos-López *et al*. 2021).

Among the methods used in GS, Ridge regression is one of the most popular, because it is quite efficient to mitigate multicollinearity (high correlation between predictor variables) and overfitting in predictive models in the context of more predictors (p) than observations (n). Ridge regression addresses these issues by introducing a regularization (penalization) parameter, often denoted as lambda (*λ*), to the standard least squares objective function (Montesinos-López *et al*. 2022). This regularization parameter penalizes large coefficient values, effectively shrinking them toward 0. The amount of shrinkage is controlled by the regularization parameter *λ*, which is a nonnegative value chosen by the analyst. However, an efficient implementation of Ridge regression needs a good selection of the regularization parameter *λ*. Through their application, these models achieve heightened efficiency and prediction accuracy, showcasing their instrumental role in contemporary data analytics. Due to this, Ridge regression is widely used in various fields, including statistics, machine learning, GS, and data analysis, particularly when dealing with datasets with multicollinearity or high-dimensional predictor spaces.

Ridge regression stands out as a crucial asset in the realm of genomic prediction, offering a versatile solution across a multitude of fields. Its adept handling of high-dimensional genomic datasets empowers researchers to extract pertinent insights, crucial to advance genetic improvement endeavors in plants, animals, and beyond. By tackling the complexities inherent in such data, Ridge regression plays a pivotal role in identifying and selecting elite individuals suited for breeding programs across diverse species. Its ability to navigate through the intricacies of genetic information underscores its significance as a fundamental tool in modern genetic research and agricultural innovation.

There is a large amount of empirical evidence that Ridge regression is a power tool to predict out-of-sample data in a quite efficient manner; however, the quality of the predictions depends, to a great extent on and adequate selection of the regularization parameter. For this reason, some methods for its optimal selection have been developed so far; some methods are better than others, although anyone of them is still optimal. For this reason, in this paper, with the goal of improving the efficiency of the Ridge regression in terms of prediction performance, we propose a novel method to select the optimal regularization parameter. The proposed method was compared with the most popular method to select the regularization parameter that comes implemented in the glmnet library (Friedman *et al*. 2010; Simon *et al*. 2011; Tay *et al*. 2023). The empirical comparison was carried out using 14 real datasets with phenotypic and markers data.

## Materials and methods

### Datasets

A concise overview of the 14 datasets used in this study is provided in Table A1 (Appendix A).

### Statistical model

In a general context, we have a covariate vector $x_{i}=(x_{i1},\;\ldots ,\;x_{ip}{)}^{T},$i=1,…,n, and we want to use this information to predict or explain how this variable affects a real-value response $y_{i}$. The linear multiple regression model assumes a relationship given by


(1) $$y_{i}=\beta_{0}+\overset{p}{\underset{\;j=1}{\sum }}\;x_{ij}\beta_{j}+\epsilon_{i}$$

where $\epsilon_{i}$ is a random error vector with mean 0, $E(\epsilon_{i})=0$ and is independent of $x_{i}$. This error is included in the model to capture measurement errors and the effects of other unregistered explanatory variables that can help explain the mean response. Then, the conditional mean of this model is $E(y_{i}\;|x_{i})=\beta_{0}+\overset{p}{\underset{\;j=1}{\sum }}\;x_{ij}\beta_{j}$ and the conditional distribution of $y_{i}$ given $x_{i}$ is only affected by the information of $x_{i}$.

To estimate the parameters $\beta =(\beta_{0},\beta_{1},\;\ldots ,\;\;\beta_{p}{)}^{T}$, we usually have a set of data $\left(x_{i}^{T},y_{i}\right)$, i=1,…,n, often known as training data, where $x_{i}$ is a vector of features measurement and $y_{i}$ is the response measurement corresponding to the *i* th individual drawn. In the context of large *p* and small *n*, the most common method to estimate β is the Ridge regression method, which consists of taking the β value that minimizes the penalized residual sum of squares (Montesinos-López *et al*. 2022) defined as


$$\mathrm{PRS}S_{\lambda }(\beta )=\overset{n}{\underset{i=1}{\sum }}\;{\left(y_{i}-\beta_{0}-\overset{p}{\underset{\;j=1}{\sum }}\;x_{ij}\beta_{j}\right)}^{2}+\lambda \overset{p}{\underset{\;j=1}{\sum }}\;\beta_{j}^{2}\;$$

where λ≥0 is the regularization parameter, which determines the level or degree to which the beta coefficients are shrunk toward 0. When λ=0, the ordinary least square (OLS) is the solution to the beta coefficients, but when *λ* is large, the $\mathrm{PRS}S_{\lambda }(\beta )$ is dominated by the penalization term, and the OLS solution must shrink toward 0 (Christensen 2011). In general, when the number of parameters to be estimated is larger than the number of observations, the estimator resulting in the OLS is invalid. In this situation, the intuition of Ridge regression tries to alleviate this by constraining the sum of squares for the beta coefficients (Wakefield 2013).

### Bayesian GBLUP

The Bayesian Genomic Best Linear Unbiased Predictor (GBLUP) model is formulated as a regression problem and is given by:


(2) $$y_{i}=\mu +g_{i}+\epsilon_{i}$$

where $y_{i}$ denotes the continues response variable measured in the *i*th line, *μ* is a general mean or intercept. $g_{j}$ (i=1,…,J), denotes the random effect of *i*th genotype, and $\epsilon_{i}$ is the random error component of *i*th genotype distributed as an independent normal random variable with mean 0 and variance $\sigma^{2}$. It is assumed that $g=(g_{1},\ldots ,g_{J}{)}^{T}∼N_{J}(0,\sigma_{g}^{2}G)$, where G is a linear kernel known as genomic relationship matrix computed according with the method of VanRaden (2008). This model was implemented in the R statistical software (R Core Team (2024)) with the BGLR library of Pérez and de Los Campos (2014). It is important to point out that this model given in Equation (2) is only a reparameterization of model given in Equation (1). Also, the model given in Equation (2) was implemented under a mixed model framework in the library rrBLUP of Endelman (2011).

### Conventional approach for tuning the lambda parameter (glmnet)

Given that there are many methods to select the regularization parameter (*λ*), in this study we will use the following cross-validation method as a reference to select this hyperparameter. We will illustrate this method by selecting 100 λ values. First, the data are divided into training and testing set. Then the training is divided into inner training and validation set. The steps according to Tay *et al*. (2023) for this procedure are as follows:

1. Step 1. Standardize the training data ($y_{\mathrm{trn}}$, $X_{\mathrm{trn}})$. We standardize the response variable ($y_{\mathrm{trn}}$) and each column of the input matrix $\left(X_{\mathrm{trn}}\right)$, but with the variance computed as $\sigma_{X_{\;j,\mathrm{trn}}}^{2}=\overset{n_{\mathrm{trn}}}{\underset{i=1}{\sum }}\;(X_{ij}-{\bar{X}}_{\;j,\mathrm{trn}}{)}^{2}/n_{\mathrm{trn}}$, where $n_{\mathrm{trn}}$ is the number of training data points and ${\bar{X}}_{\;j,\mathrm{trn}}$ is the average of the column *j* of the matrix $X_{\mathrm{strn}}.$ That is, each column of $X_{\mathrm{strn}}$ is standardized as: $X_{\;j,\mathrm{strn}}=\frac{X_{ij}-{\bar{X}}_{j,\mathrm{trn}}}{\sigma_{X_{\;j,\mathrm{trn}}}}$
2. Step 2. We collect the standardized training data in ($y_{\mathrm{strn}}$, $X_{\mathrm{strn}}$) by subtracting from it its sample mean and by dividing it by its standard deviation (SD).
  $$y_{\mathrm{trn}}=(y_{1},\;\ldots ,\;y_{n_{\mathrm{trn}}}{)}^{T}$$
  $${\bar{y}}_{\mathrm{trn}}=\frac{\sum_{i=1}^{n_{\mathrm{trn}}}\;y_{i}}{n_{\mathrm{trn}}}$$
  $$\sigma_{y_{\mathrm{trn}}}=\sqrt{\overset{n_{\mathrm{trn}}}{\underset{i=1}{\sum }}\;{\left(y_{i}-{\bar{y}}_{\mathrm{trn}}\right)}^{2}/n_{\mathrm{trn}}}$$
  $$y_{\mathrm{strn}}=\frac{y_{\mathrm{trn}}-{\bar{y}}_{\mathrm{trn}}}{\sigma_{y_{\mathrm{trn}}}}$$
3. Step 3. We compute the element-wise product of each column of $X_{\mathrm{strn}}$ with $y_{\mathrm{strn}},\;\mathrm{and}\;\mathrm{this}\;\mathrm{information}\;\mathrm{is}\;\mathrm{saved}\;\mathrm{in}\;\;P_{XY}$, where $X_{\mathrm{strn}}\;\epsilon \;R^{n_{\mathrm{trn}}\times p}$, $y_{\mathrm{strn}}\;\epsilon \;R^{n_{\mathrm{trn}}\times 1}$ and $P_{XY}\;\epsilon \;R^{n_{\mathrm{trn}}\times p}$
4. Step 4. Next, we compute the sum of each column of $(P_{XY}\times 1000)/n_{\mathrm{trn}}$ and this sum of columns is saved in $S_{XY}.$
5. Step 5. We compute $\lambda_{\max}=\max(S_{XY})$
6. Step 6. Next, we define the $\lambda_{\min.\mathrm{ratio}}=0.01\;$ if number of predictors (p) is larger than the number of observations ($n_{\mathrm{trn}}$); otherwise the $\lambda_{\min.\mathrm{ratio}}=0.0001$.
7. Step 7. We compute $\lambda_{\min}=\lambda_{\max}\times \lambda_{\min.\mathrm{ratio}}$.
8. Step 8. Next, we generate 100 λ values equally spaced between the $\log(\lambda_{\max})$ and $\mathrm{loglog}\;(\;\lambda_{\min})$. These 100 λ values can be generated as $\log(\;\lambda_{l})=\log(\;\lambda_{\min})+\left[\frac{\left[\log(\lambda_{\max})-\log\;(\;\lambda_{\min})\right]}{99}\right]\times (l-1)$, l=1,…,100.
9. Step 9. Then, with 10-fold cross-validation, we divide the training in inner training and validation and with the inner training is trained, the model for each of the 100 regularization parameters and its prediction error is evaluated in the validation set and as optimal lambda is chosen, the one that provide less MSE in the average of the 10-folds.

In the glmnet library, in which the models will be implemented, this method of tuning is provided by default but not necessary; in the tuning process, the 100 values of lambda are evaluated, since according to the default internal settings, the computations stop if either the fractional change in deviance down the path is less than 1 × 10^−5^ or if the fraction of explained deviance reaches 0.999.

For example, in the scikit-learn Python library, the ElasticNetCV function facilitates the implementation of Lasso, Ridge, and Elastic Net regression. By default, ElasticNetCV generates 100 values for the regularization parameter, lambda, which range from 10^–4^ to 10 and are spaced logarithmically. Additionally, it's important to note that this library allows providing customized values for lambda.

### Proposed approach for tuning the regularization hyperparameter (glmnet M)

From a mixed (or Bayesian) model framework, *λ* is estimated as a ratio of variance components as $\lambda =\sigma^{2}/\sigma_{\beta }^{2}$, where $\sigma^{2}$ is the variance of the error term and $\sigma_{\beta }^{2}$ is the variance of the beta coefficients, which guarantees a lower mean squared error in future predicted values (Montesinos-López *et al*. 2022). However, under penalized Ridge regression, *λ* is typically chosen by cross-validation with the training set. This can be done with the default conventional approach explained above with the grid search method implemented in the cv.glmnet function of the glmnet R package (Friedman *et al*. 2010).

With the goal of proposing a better approach to select the optimal regularization parameter (λ), we propose to use the same cv.glmnet function but specifying a custom grid of values of *λ*. The approach explores approximately different proportion values of phenotypic variance $\left(R_{l}^{2}\right)$ that the genotypic effects ($x_{i}^{T}\beta$ ) can explain, starting from a small value ($10^{-5}$) up to a large value (0.999). Particularly, the explored grid values of *λ* are given by


(2) $$\begin{array}{rl} \lambda_{l} & =\frac{\sigma_{l}^{2}}{\sigma_{l\beta }^{2}}=\frac{(1-R_{l}^{2})s_{y}^{2}}{R_{l}^{2}s_{y}^{2}/\left(\frac{1}{n_{\mathrm{trn}}}\sum_{i=1}^{n_{\mathrm{trn}}}\;x_{i}^{T}x_{i}\right)}=\frac{1-R_{l}^{2}}{R_{l}^{2}/\left(\frac{1}{n_{\mathrm{trn}}}\sum_{i=1}^{n_{\mathrm{trn}}}\;x_{i}^{T}x_{i}\right)},\\ l & =1,\ldots ,100 \end{array}$$

where $s_{y}^{2}$ represent the phenotypic variance in the training data, and


$$\sigma_{l\beta }^{2}=\frac{R_{l}^{2}s_{y}^{2}}{\left(\frac{1}{n_{\mathrm{trn}}}\sum_{i=1}^{n_{\mathrm{trn}}}\;x_{i}^{T}x_{i}\right)}$$

Denotes a proportion $R_{l}^{2}$ of the genotypic variance explained by the $x_{i}^{T}\beta$ term (genotypic effects) under method (1) (Montesinos-López *et al*. 2022; see details in Appendix A). $\sigma_{l}^{2}=(1-R_{l}^{2})s_{y}^{2}$ represents the remaining proportion of the phenotypic variance left to the variance error, where


(3) $$R_{l}^{2}=\exp(lR_{l})\;$$

and


(4) $$lR_{l}=\log\;(10^{-5})\;+\left[\frac{\left[\log\;(0.9999)\;-\log\;(10^{-5})\;\right]}{99}\right]\times (l-1),\;$$

l=1,…,100 , are the different proportions of phenotypic variance explained by the genotypic effects to be explored. For each value of *λ* in this grid, the average performance prediction, measured by the mean square error (MSE computed as $\overset{n_{\mathrm{val}}}{\underset{i=1}{\sum }}\;(y_{i}-\hat{y_{i}}{)}^{2}$ where $n_{\mathrm{val}}$ denotes the number of observations in the validation set and $\hat{y_{i}}$ denotes the predicted value i) obtained across an inner 10-fold cross-validation strategy, is calculated. Then, the value of *λ* that corresponds to the smallest MSE in this grid in the validation data, is chosen as the optimal *λ* value. Subsequently, the model is fitted with the entire training set using this optimal value, which is then evaluated on the testing set.

This proposed method for selecting the grid of values does not guarantee perfect results in every case. However, it is effective because it selects the grid values by computing the ratio of the variance components of the error ($\sigma^{2}$) and inputs ($\sigma_{\beta }^{2}$), and for this reason has more chance to provide a more optimal result. More optimal results are expected because the derivation of the grid values considers both the inputs and the response variable of the available training set, that is, use prior information of the training in its derivation (See Equation A1, in Appendix A). Additionally, to enhance the efficacy of the proposed method, it is important to consider using a grid with more than 100 values.

### Outer cross-validation strategy

For the comparison of the proposed and conventional models, we used cross-validation. We implemented a 10-Fold Cross-Validation, dividing the dataset into 10 similarly sized subsets, using 9 of them for training and 1 for testing, and repeating this process 10 times (once for each subset as the test set). Then, for each testing set the accuracy was computed in terms of average Pearson's correlation (Cor) and normalized root mean square error (NRMSE) (Montesinos-López *et al*. 2022). $\mathrm{MSE}=\frac{1}{n_{\mathrm{tst}}}(\overset{n_{t\mathrm{st}}}{\underset{i=1}{\sum }}\;(y_{i}-\hat{\;f}(x_{i}){)}^{2}$, where MSE denotes the mean square error, $y_{i}$ denotes the observed value of the *i*th observation, $n_{\mathrm{tst}}$ denotes the size of the testing set and $\hat{\;f}(x_{i})$ is the prediction that $\hat{\;f}$ gives to the *i*th observation. Then $\mathrm{NRMSE}=\frac{\sqrt{\mathrm{MSE}}}{\sum_{i=1}^{n_{\mathrm{tst}}}\;\frac{y_{i}}{n_{\mathrm{tst}}}}$. We used the NRMSE since this metrics allows the comparison of results between different traits since not depend on the scale of the trait. For the computation of both metrics we used the observed values ($y_{i}$) and predicted values [ $\hat{\;f}(x_{i})$ ] in each fold (partition) corresponding to the testing set. The average performance across the 10 folds was reported using these metrics. It is important to point out that we used outer cross-validation to differentiate the inner cross-validation that was used for tuning the regularization parameter (*λ*) in which each outer training set was divided into inner training and validation set. For the inner cross-validation, we used 10-fold cross-validation. We used Cor and NRMSE since are 2 popular metrics of prediction performance in genomic prediction and of course, more metrics exist but for the goal of the paper these 2 metrics we consider enough and appropriate.

## Results

**Fig. 1.**
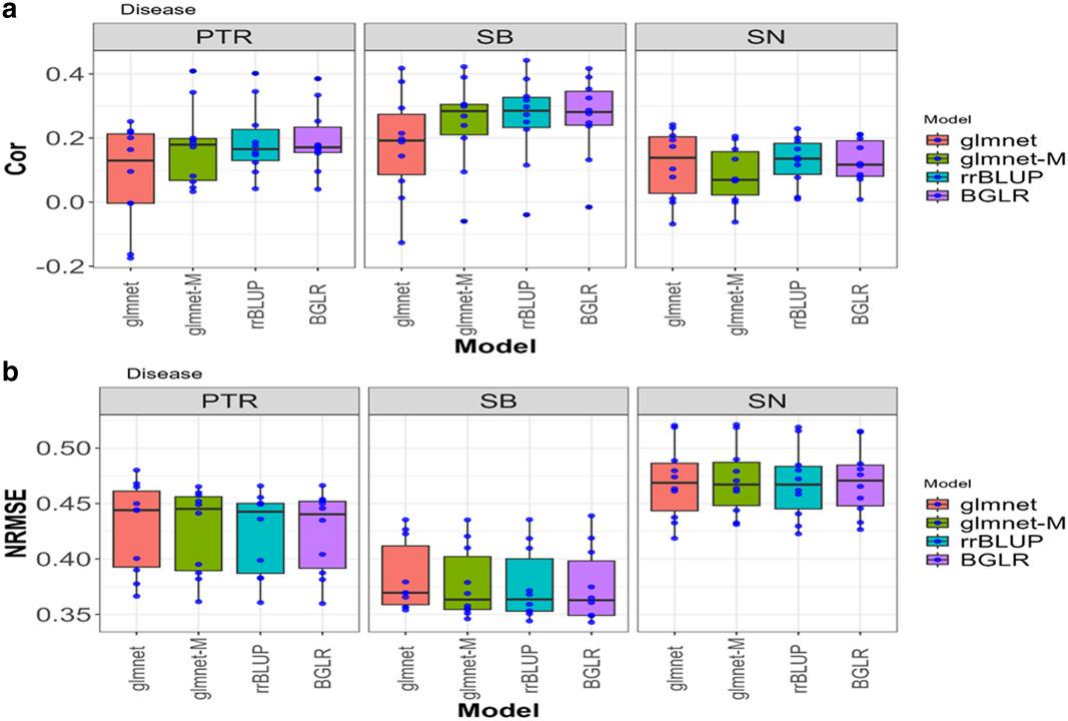
Box plots for the *disease* dataset with glmnet and gmlnet-M methods. a) Boxplot of the performance with Cor between the observed and predicted values through a 10-fold cross-validation for each of the 3 traits (PTR, SB, and SN). b) Box plot of the performance with the NRMSE between the observed and predicted values through a 10-fold cross-validation for each of the 3 traits (PTR, SB, and SN).

**Fig. 2.**
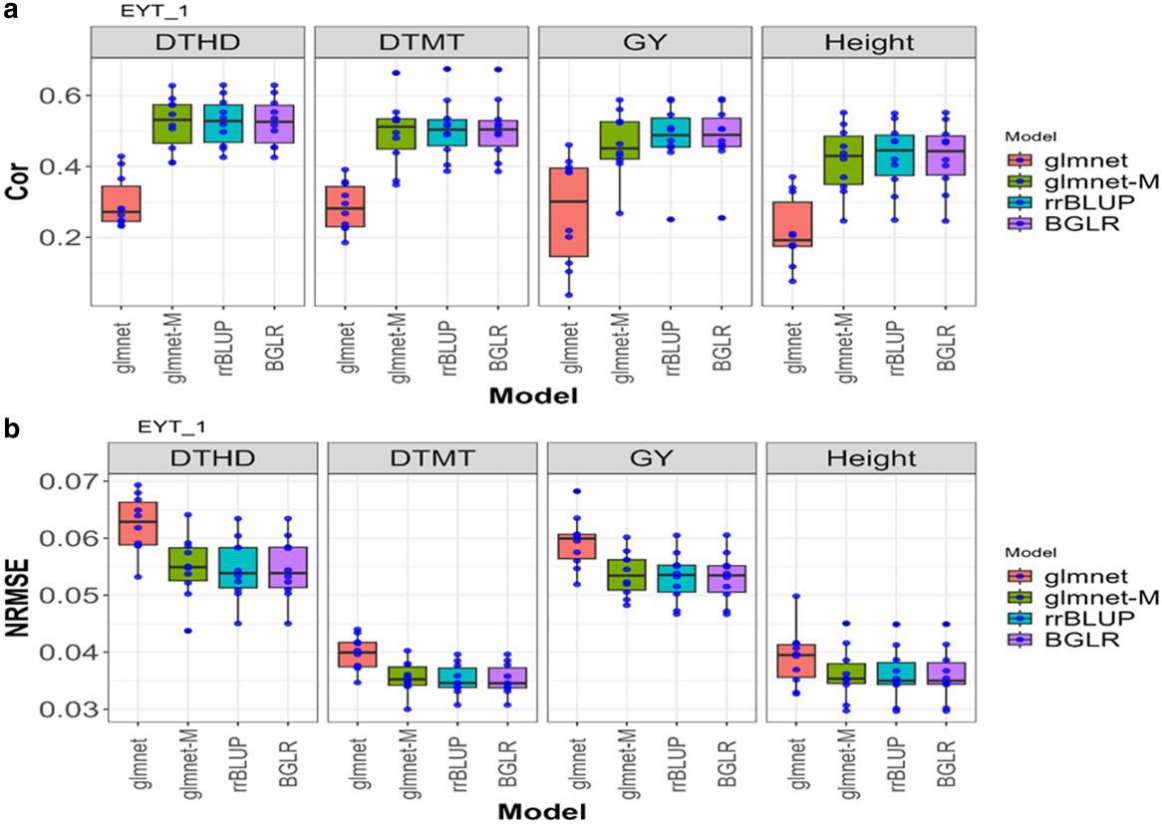
Box plots for the *EYT_1* dataset with glmnet and gmlnet-M methods. a) Box Plot of the performance with Cor between observed and predicted values through 10-fold cross-validation for each of the 4 traits (DTHD, DTMT, GY, and Height). b) Box Plot of the performance with the NRMSE between observed and predicted values through a 10-fold cross-validation for each of the 4 traits (DTHD, DTMT, GY, and Height).

**Fig. 3.**
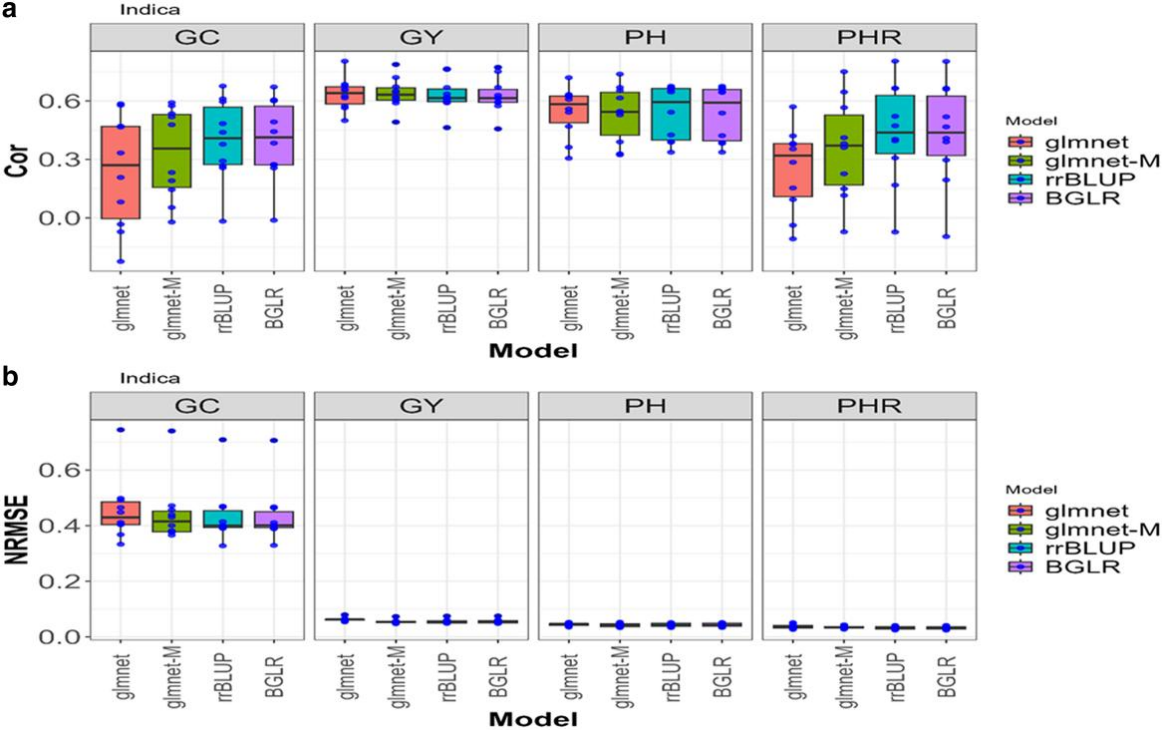
Box plots for the *Indica* dataset with glmnet and gmlnet-M methods. a) Box Plot of the performance with Cor between observed and predicted values through 10-fold cross-validation for each of the 4 traits (GC, GY, PH, and PHR). b) Box Plot of the performance with the NRMSE between observed and predicted values through a 10-fold cross-validation for each of the 4 traits (GC, GY, PH, and PHR).

**Fig. 4.**
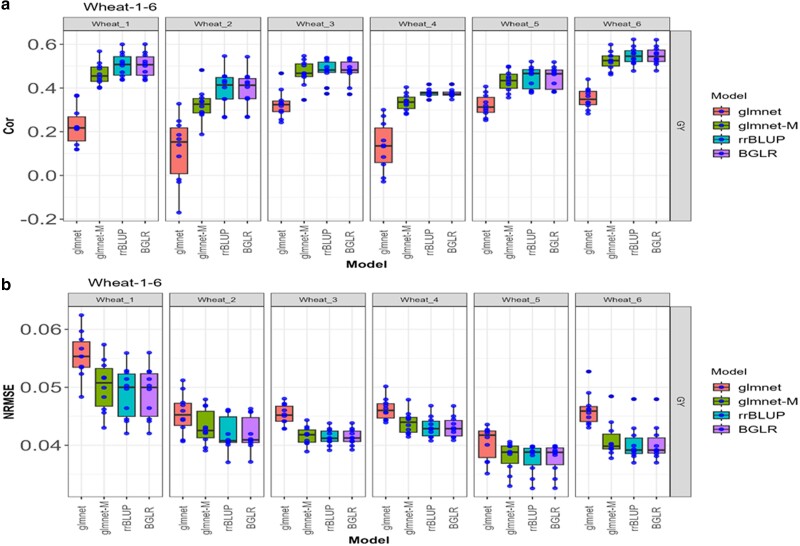
Box plots for each of the *Wheat_1-Wheat_6* datasets with glmnet and gmlnet-M methods. a) Box Plot of the performance with Cor between observed and predicted values through 10-fold cross-validation for the unique trait (GY). b) Box Plot of the performance with the NRMSE between observed and predicted values through a 10-fold cross-validation for the unique trait (GY).

**Fig. 5.**
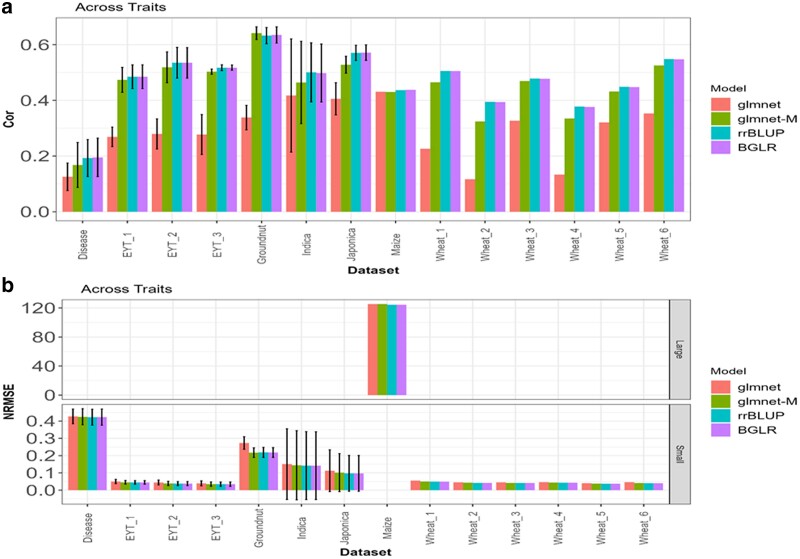
a) average Cor *across traits* of the mean values obtained across folds, with glmnet and glmnet-M methods in each dataset (dataset). The limits of the vertical lines in each bar indicate the average minus and plus 1 SD values of the correlation obtained across traits. b) Average NRMSE across traits of the mean values obtained across folds, with glmnet and glmnet-M methods in each dataset (Dataset). The limits of the vertical lines in each bar indicate the average minus and plus 1 SD values of the NRMSE obtained across traits.

**Fig. B1.**
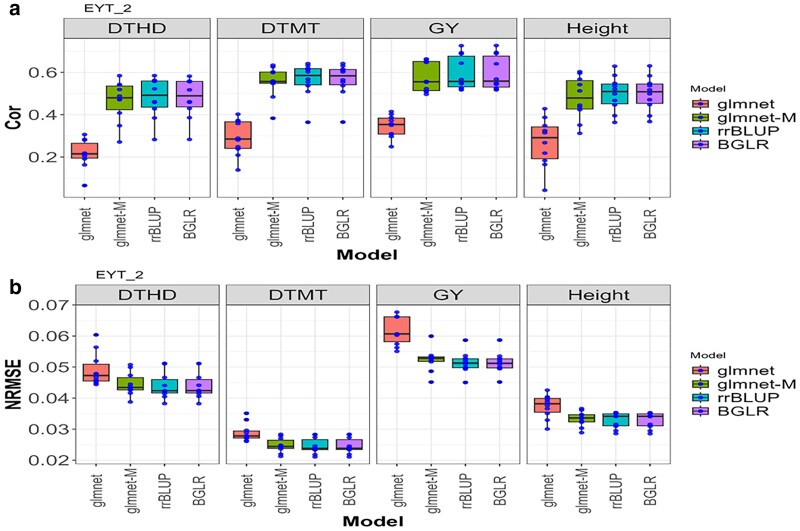
Box plots for the EYT_2 dataset with glmnet and gmlnet-M Models (model). a) Box Plot of the performance with Cor between observed and predicted values through 10-fold cross-validation for each of the 4 traits (DTHD, DTMT, GY, and Height). b) Box Plot of the performance with the NRMSE between observed and predicted values through 10-fold cross-validation for each of the 4 traits (DTHD, DTMT, GY, and Height).

**Fig. B2.**
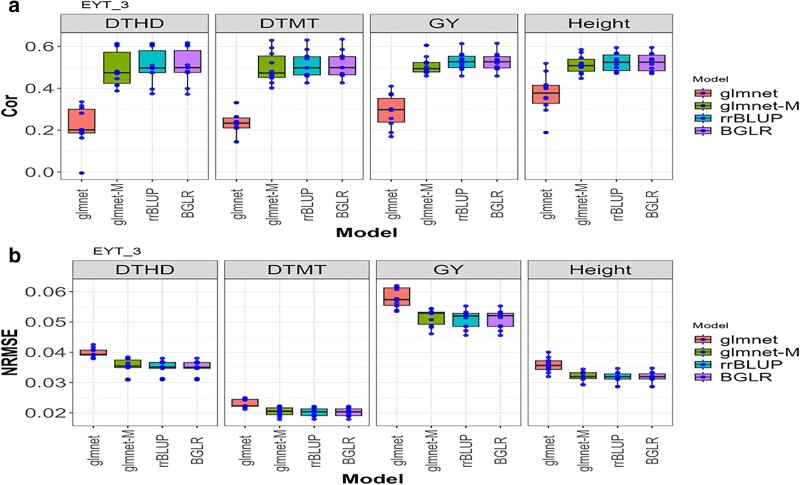
Box plots for the EYT_3 dataset with glmnet and gmlnet-M Models (model). a) Box Plot of the performance with Cor between observed and predicted values through 10-fold cross-validation for each of the 4 traits (DTHD, DTMT, GY, and Height). b) Box Plot of the performance with the NRMSE between observed and predicted values through 10-fold cross-validation for each of the 4 traits (DTHD, DTMT, GY, and Height).

**Fig. B3.**
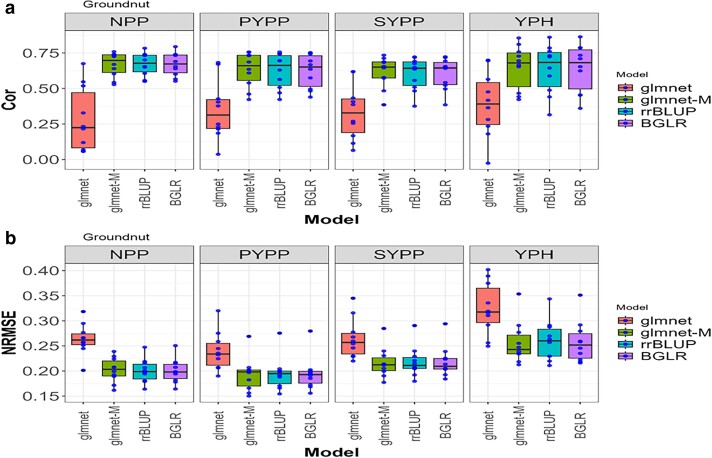
Box plots for the *Groundnut* dataset with glmnet and gmlnet-M Models (model). a) Box Plot of the performance with Cor between observed and predicted values through 10-fold cross-validation for each of the 4 traits (NPP, PYPP, SYPP, and YPH). b) Box Plot of the performance with the NRMSE between observed and predicted values through 10-fold cross-validation for each of the 4 traits (NPP, PYPP, SYPP, and YPH).

**Fig. B4.**
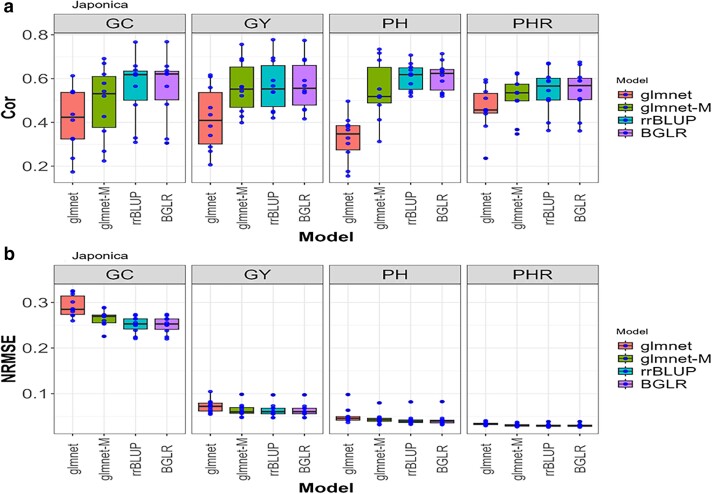
Box plots for the *Japonica* dataset with glmnet and gmlnet-M Models (model). a) Box Plot of the performance with Cor between observed and predicted values through 10-fold cross-validation for each of the 4 traits (GC, GY, PH, and PHR). b) Box Plot of the performance with the NRMSE between observed and predicted values through 10-fold cross-validation for each of the 4 traits (GC, GY, PH, and PHR).

**Fig. B5.**
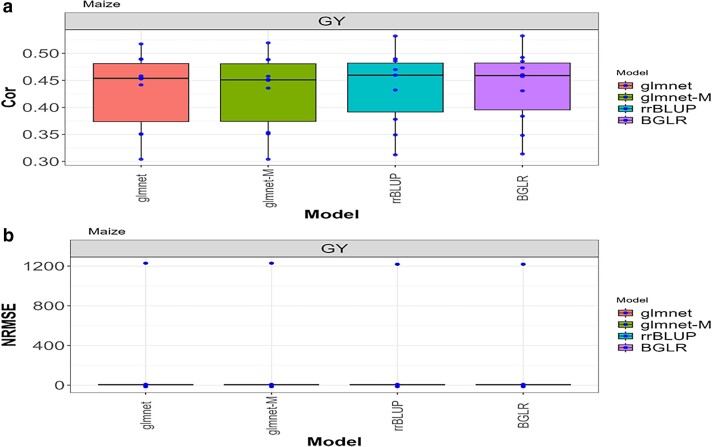
Box plots for the *Maize* dataset with glmnet and gmlnet-M Models (model). a) Box Plot of the performance with Cor between observed and predicted values through 10-fold cross-validation for the unique trait (GY). b) Box Plot of the performance with the NRMSE between observed and predicted values through 10-fold cross-validation for the unique trait (GY).

The results presented in this section provide a detailed comparison of the performance between the conventional tuning process of the regularization parameter (*λ*), denoted as “glment,” since it was implemented in this library, and the proposed novel tuning process denoted as “glmnet-M,” since we modified the tuning process given as default in the glmnet library. The comparison was carried out using 14 datasets: Disease, EYT_1, EYT_2, EYT_3, Groundnut, Indica, Japonica, Maize, Wheat_1-Wheat_6; see Table A1 (Appendix A). Additionally, an “across dataset” evaluation is presented. Note that this section provides the results for datasets Disease (Fig. 1), EYT_1 (Fig. 2), Indica (Fig. 3), Wheat_1-Wheat_6 (Fig. 4) and “across datasets” (Fig. 5). The remaining results are in Appendix B for datasets EYT_2 (Fig. B1), EYT_3 (Fig. B2), Groundnut (Fig. B3), Japonica (Fig. B4) and maize (Fig. B5).

Furthermore, summarizes of the Average Normalized Root Mean Squared Error (NRMSE) and average Cor across the 10-folds, with glmnet and glmnet-M methods can be found for: (1) each dataset (Dataset) and for each trait (Trait) (Table 1), (2) across traits and (3) across folds (Table 2), and (4) across different datasets (Dataset) of the mean values obtained across traits (Table 3).

**Table 1.** Average NRMSE and average Cor across the 10-folds, with glmnet, glmnet-M, rrBLUP, and BGLR methods in each dataset (dataset) and for each trait (trait).

Method	Dataset	Trait	NRMSE (SD)	Cor (SD)
glmnet	Disease_AL	PTR	0.4285 (0.041)	0.0806 (0.1593)
glmnet-M	Disease_AL	PTR	0.425 (0.0389)	0.1726 (0.1257)
rrBLUP	Disease_AL	PTR	0.4231 (0.0378)	0.1912 (0.1106)
BGLR	Disease_AL	PTR	0.4236 (0.0371)	0.1941 (0.1038)
glmnet	Disease_AL	SB	0.3835 (0.0318)	0.1783 (0.1652)
glmnet-M	Disease_AL	SB	0.3777 (0.0323)	0.2464 (0.1416)
rrBLUP	Disease_AL	SB	0.3762 (0.0326)	0.2598 (0.1375)
BGLR	Disease_AL	SB	0.3754 (0.0339)	0.2648 (0.1282)
glmnet	Disease_AL	SN	0.4694 (0.0343)	0.1171 (0.1092)
glmnet-M	Disease_AL	SN	0.4711 (0.0318)	0.0852 (0.0892)
rrBLUP	Disease_AL	SN	0.4684 (0.0328)	0.1272 (0.0748)
BGLR	Disease_AL	SN	0.4697 (0.0308)	0.1269 (0.0688)
glmnet	EYT_1_AL	DTHD	0.0624 (0.005)	0.2985 (0.074)
glmnet-M	EYT_1_AL	DTHD	0.0549 (0.0055)	0.521 (0.0758)
rrBLUP	EYT_1_AL	DTHD	0.0546 (0.0054)	0.5259 (0.0686)
BGLR	EYT_1_AL	DTHD	0.0547 (0.0054)	0.5256 (0.0685)
glmnet	EYT_1_AL	DTMT	0.0397 (0.003)	0.2857 (0.0677)
glmnet-M	EYT_1_AL	DTMT	0.0355 (0.0028)	0.4933 (0.0937)
rrBLUP	EYT_1_AL	DTMT	0.0352 (0.0026)	0.5053 (0.0847)
BGLR	EYT_1_AL	DTMT	0.0352 (0.0027)	0.5053 (0.084)
glmnet	EYT_1_AL	GY	0.0593 (0.0046)	0.2732 (0.1524)
glmnet-M	EYT_1_AL	GY	0.0537 (0.0038)	0.4625 (0.0929)
rrBLUP	EYT_1_AL	GY	0.0531 (0.0043)	0.4809 (0.0968)
BGLR	EYT_1_AL	GY	0.053 (0.0043)	0.4821 (0.0956)
glmnet	EYT_1_AL	Height	0.039 (0.005)	0.2179 (0.0977)
glmnet-M	EYT_1_AL	Height	0.0362 (0.0046)	0.417 (0.0949)
rrBLUP	EYT_1_AL	Height	0.036 (0.0046)	0.4274 (0.0966)
BGLR	EYT_1_AL	Height	0.036 (0.0046)	0.4267 (0.0962)
glmnet	EYT_2_AL	DTHD	0.0491 (0.0053)	0.2141 (0.0692)
glmnet-M	EYT_2_AL	DTHD	0.0445 (0.0037)	0.4644 (0.0966)
rrBLUP	EYT_2_AL	DTHD	0.044 (0.0043)	0.48 (0.0962)
BGLR	EYT_2_AL	DTHD	0.044 (0.0043)	0.4789 (0.0947)
glmnet	EYT_2_AL	DTMT	0.0289 (0.0029)	0.2905 (0.0845)
glmnet-M	EYT_2_AL	DTMT	0.0248 (0.0023)	0.5537 (0.0746)
rrBLUP	EYT_2_AL	DTMT	0.0245 (0.0025)	0.564 (0.0825)
BGLR	EYT_2_AL	DTMT	0.0246 (0.0025)	0.5628 (0.0816)
glmnet	EYT_2_AL	GY	0.0616 (0.0046)	0.3447 (0.0527)
glmnet-M	EYT_2_AL	GY	0.0523 (0.0037)	0.5773 (0.0706)
rrBLUP	EYT_2_AL	GY	0.0513 (0.0034)	0.5974 (0.0811)
BGLR	EYT_2_AL	GY	0.0513 (0.0034)	0.5974 (0.0815)
glmnet	EYT_2_AL	Height	0.0373 (0.0037)	0.2679 (0.1167)
glmnet-M	EYT_2_AL	Height	0.0333 (0.0024)	0.4787 (0.1014)
rrBLUP	EYT_2_AL	Height	0.0328 (0.0025)	0.4989 (0.0827)
BGLR	EYT_2_AL	Height	0.0328 (0.0025)	0.4995 (0.0818)
glmnet	EYT_3_AL	DTHD	0.0398 (0.0015)	0.2176 (0.1001)
glmnet-M	EYT_3_AL	DTHD	0.0353 (0.0026)	0.4918 (0.0859)
rrBLUP	EYT_3_AL	DTHD	0.035 (0.0023)	0.505 (0.0837)
BGLR	EYT_3_AL	DTHD	0.0349 (0.0023)	0.5064 (0.0841)
glmnet	EYT_3_AL	DTMT	0.023 (0.0013)	0.2281 (0.0559)
glmnet-M	EYT_3_AL	DTMT	0.0204 (0.0014)	0.499 (0.0751)
rrBLUP	EYT_3_AL	DTMT	0.0202 (0.0014)	0.512 (0.0642)
BGLR	EYT_3_AL	DTMT	0.0202 (0.0014)	0.5134 (0.0644)
glmnet	EYT_3_AL	GY	0.0579 (0.0032)	0.2896 (0.0797)
glmnet-M	EYT_3_AL	GY	0.0514 (0.0028)	0.5081 (0.044)
rrBLUP	EYT_3_AL	GY	0.0508 (0.0031)	0.5278 (0.044)
BGLR	EYT_3_AL	GY	0.0508 (0.0031)	0.5273 (0.0439)
glmnet	EYT_3_AL	Height	0.0357 (0.0024)	0.3735 (0.0944)
glmnet-M	EYT_3_AL	Height	0.0319 (0.0017)	0.5128 (0.0444)
rrBLUP	EYT_3_AL	Height	0.0316 (0.0019)	0.524 (0.0433)
BGLR	EYT_3_AL	Height	0.0316 (0.0019)	0.5231 (0.0436)
glmnet	Groundnut_AL	NPP	0.2633 (0.0309)	0.2823 (0.2266)
glmnet-M	Groundnut_AL	NPP	0.2022 (0.0246)	0.6679 (0.0856)
rrBLUP	Groundnut_AL	NPP	0.2005 (0.0237)	0.6711 (0.0807)
BGLR	Groundnut_AL	NPP	0.2005 (0.0242)	0.6708 (0.0815)
glmnet	Groundnut_AL	PYPP	0.2395 (0.0381)	0.348 (0.2086)
glmnet-M	Groundnut_AL	PYPP	0.1931 (0.0337)	0.6334 (0.1233)
rrBLUP	Groundnut_AL	PYPP	0.1949 (0.0329)	0.623 (0.1239)
BGLR	Groundnut_AL	PYPP	0.1948 (0.0335)	0.6235 (0.1186)
glmnet	Groundnut_AL	SYPP	0.2645 (0.0397)	0.3335 (0.1941)
glmnet-M	Groundnut_AL	SYPP	0.2167 (0.0299)	0.6141 (0.1103)
rrBLUP	Groundnut_AL	SYPP	0.2186 (0.0306)	0.6019 (0.1158)
BGLR	Groundnut_AL	SYPP	0.2185 (0.031)	0.6026 (0.111)
glmnet	Groundnut_AL	YPH	0.3245 (0.0522)	0.389 (0.2312)
glmnet-M	Groundnut_AL	YPH	0.2562 (0.0418)	0.6497 (0.1553)
rrBLUP	Groundnut_AL	YPH	0.2603 (0.0404)	0.6338 (0.1733)
BGLR	Groundnut_AL	YPH	0.2578 (0.0415)	0.6427 (0.1682)
glmnet	Indica_AL	GC	0.4568 (0.1139)	0.2396 (0.2903)
glmnet-M	Indica_AL	GC	0.4439 (0.1105)	0.3299 (0.2335)
rrBLUP	Indica_AL	GC	0.4367 (0.1038)	0.3984 (0.2086)
BGLR	Indica_AL	GC	0.4358 (0.1027)	0.3986 (0.208)
glmnet	Indica_AL	GY	0.063 (0.0065)	0.636 (0.0825)
glmnet-M	Indica_AL	GY	0.0549 (0.0069)	0.6392 (0.0793)
rrBLUP	Indica_AL	GY	0.0552 (0.0078)	0.6313 (0.0874)
BGLR	Indica_AL	GY	0.0554 (0.008)	0.627 (0.0899)
glmnet	Indica_AL	PH	0.0451 (0.0054)	0.5447 (0.1294)
glmnet-M	Indica_AL	PH	0.0425 (0.0068)	0.5334 (0.1444)
rrBLUP	Indica_AL	PH	0.0426 (0.0066)	0.5395 (0.1393)
BGLR	Indica_AL	PH	0.0428 (0.0066)	0.5362 (0.1394)
glmnet	Indica_AL	PHR	0.0369 (0.0078)	0.2493 (0.2163)
glmnet-M	Indica_AL	PHR	0.0349 (0.0048)	0.3537 (0.2553)
rrBLUP	Indica_AL	PHR	0.0331 (0.0055)	0.433 (0.2577)
BGLR	Indica_AL	PHR	0.0331 (0.0055)	0.4308 (0.2598)
glmnet	Japonica_AL	GC	0.2921 (0.0236)	0.4134 (0.146)
glmnet-M	Japonica_AL	GC	0.2652 (0.0185)	0.49 (0.1639)
rrBLUP	Japonica_AL	GC	0.2512 (0.0184)	0.5604 (0.146)
BGLR	Japonica_AL	GC	0.251 (0.0186)	0.5604 (0.1478)
glmnet	Japonica_AL	GY	0.0726 (0.0146)	0.4172 (0.1452)
glmnet-M	Japonica_AL	GY	0.0641 (0.0148)	0.5594 (0.1192)
rrBLUP	Japonica_AL	GY	0.0635 (0.0146)	0.5704 (0.1182)
BGLR	Japonica_AL	GY	0.0634 (0.0145)	0.5717 (0.1152)
glmnet	Japonica_AL	PH	0.0515 (0.0178)	0.3265 (0.1055)
glmnet-M	Japonica_AL	PH	0.0451 (0.0133)	0.5443 (0.1345)
rrBLUP	Japonica_AL	PH	0.0432 (0.0142)	0.6076 (0.0626)
BGLR	Japonica_AL	PH	0.043 (0.0145)	0.6093 (0.0666)
glmnet	Japonica_AL	PHR	0.0342 (0.0034)	0.465 (0.1046)
glmnet-M	Japonica_AL	PHR	0.0312 (0.0032)	0.5178 (0.0954)
rrBLUP	Japonica_AL	PHR	0.0304 (0.0034)	0.5434 (0.1035)
BGLR	Japonica_AL	PHR	0.0304 (0.0034)	0.544 (0.1042)
glmnet	Maize_AL	GY	125.4845 (387.7244)	0.4308 (0.0708)
glmnet-M	Maize_AL	GY	125.49 (387.724)	0.43 (0.0702)
rrBLUP	Maize_AL	GY	124.4375 (384.3821)	0.4368 (0.0692)
BGLR	Maize_AL	GY	124.4613 (384.4521)	0.4377 (0.0688)
glmnet	Wheat_1_AL	GY	0.0555 (0.0039)	0.2262 (0.0898)
glmnet-M	Wheat_1_AL	GY	0.0502 (0.0044)	0.4648 (0.0529)
rrBLUP	Wheat_1_AL	GY	0.0489 (0.0045)	0.5055 (0.0551)
BGLR	Wheat_1_AL	GY	0.0489 (0.0045)	0.5052 (0.0551)
glmnet	Wheat_2_AL	GY	0.0454 (0.0034)	0.1167 (0.1509)
glmnet-M	Wheat_2_AL	GY	0.0431 (0.003)	0.3242 (0.076)
rrBLUP	Wheat_2_AL	GY	0.0419 (0.003)	0.3948 (0.0875)
BGLR	Wheat_2_AL	GY	0.0419 (0.003)	0.3937 (0.0854)
glmnet	Wheat_3_AL	GY	0.0452 (0.0017)	0.3268 (0.0658)
glmnet-M	Wheat_3_AL	GY	0.0416 (0.0015)	0.4696 (0.0592)
rrBLUP	Wheat_3_AL	GY	0.0414 (0.0014)	0.4781 (0.0543)
BGLR	Wheat_3_AL	GY	0.0414 (0.0014)	0.4773 (0.0548)
glmnet	Wheat_4_AL	GY	0.0462 (0.0019)	0.1334 (0.113)
glmnet-M	Wheat_4_AL	GY	0.0438 (0.0019)	0.3349 (0.0399)
rrBLUP	Wheat_4_AL	GY	0.0431 (0.0018)	0.3775 (0.0188)
BGLR	Wheat_4_AL	GY	0.0431 (0.0018)	0.3761 (0.0186)
glmnet	Wheat_5_AL	GY	0.0404 (0.0029)	0.321 (0.0511)
glmnet-M	Wheat_5_AL	GY	0.038 (0.0025)	0.4318 (0.0489)
rrBLUP	Wheat_5_AL	GY	0.0376 (0.0025)	0.4486 (0.0517)
BGLR	Wheat_5_AL	GY	0.0377 (0.0025)	0.4475 (0.0509)
glmnet	Wheat_6_AL	GY	0.0461 (0.0028)	0.3528 (0.0482)
glmnet-M	Wheat_6_AL	GY	0.041 (0.0031)	0.5253 (0.0424)
rrBLUP	Wheat_6_AL	GY	0.0403 (0.0031)	0.548 (0.042)
BGLR	Wheat_6_AL	GY	0.0403 (0.0031)	0.5476 (0.0421)

SD represents the standard deviation across folds, with glmnet and glmnet-M methods in each dataset (Dataset) and for each trait (Trait).

**Table 2.** Average NRMSE and average Cor across traits and across folds, with glmnet, glmnet-M, rrBLUP, and BGLR methods in each dataset (dataset).

Method	Dataset	NRMSE (SD)	Cor (SD)
glmnet	Disease_AL	0.4271 (0.0429)	0.1253 (0.0493)
glmnet-M	Disease_AL	0.4246 (0.0466)	0.1681 (0.0806)
rrBLUP	Disease_AL	0.4226 (0.046)	0.1927 (0.0662)
BGLR	Disease_AL	0.4229 (0.0471)	0.1953 (0.0689)
glmnet	EYT_1_AL	0.0501 (0.0124)	0.2688 (0.0354)
glmnet-M	EYT_1_AL	0.045 (0.0106)	0.4735 (0.0445)
rrBLUP	EYT_1_AL	0.0447 (0.0105)	0.4849 (0.0424)
BGLR	EYT_1_AL	0.0447 (0.0105)	0.4849 (0.0426)
glmnet	EYT_2_AL	0.0442 (0.0142)	0.2793 (0.054)
glmnet-M	EYT_2_AL	0.0387 (0.0121)	0.5185 (0.0554)
rrBLUP	EYT_2_AL	0.0382 (0.0118)	0.5351 (0.0549)
BGLR	EYT_2_AL	0.0382 (0.0118)	0.5347 (0.0549)
glmnet	EYT_3_AL	0.0391 (0.0144)	0.2772 (0.0716)
glmnet-M	EYT_3_AL	0.0348 (0.0128)	0.5029 (0.0093)
rrBLUP	EYT_3_AL	0.0344 (0.0126)	0.5172 (0.0105)
BGLR	EYT_3_AL	0.0344 (0.0126)	0.5175 (0.0094)
glmnet	Groundnut_AL	0.2729 (0.0362)	0.3382 (0.044)
glmnet-M	Groundnut_AL	0.217 (0.0278)	0.6413 (0.0229)
rrBLUP	Groundnut_AL	0.2185 (0.0295)	0.6325 (0.029)
BGLR	Groundnut_AL	0.2179 (0.0284)	0.6349 (0.0289)
glmnet	Indica_AL	0.1505 (0.2045)	0.4174 (0.2032)
glmnet-M	Indica_AL	0.1441 (0.2)	0.464 (0.1479)
rrBLUP	Indica_AL	0.1419 (0.1967)	0.5006 (0.1058)
BGLR	Indica_AL	0.1418 (0.1961)	0.4981 (0.104)
glmnet	Japonica_AL	0.1126 (0.1206)	0.4056 (0.0576)
glmnet-M	Japonica_AL	0.1014 (0.11)	0.5279 (0.0305)
rrBLUP	Japonica_AL	0.0971 (0.1036)	0.5704 (0.0271)
BGLR	Japonica_AL	0.097 (0.1036)	0.5714 (0.0277)
glmnet	Maize_AL	125.4845 (0)	0.4308 (0)
glmnet-M	Maize_AL	125.49 (0)	0.43 (0)
rrBLUP	Maize_AL	124.4375 (0)	0.4368 (0)
BGLR	Maize_AL	124.4613 (0)	0.4377 (0)
glmnet	Wheat_1_AL	0.0555 (0)	0.2262 (0)
glmnet-M	Wheat_1_AL	0.0502 (0)	0.4648 (0)
rrBLUP	Wheat_1_AL	0.0489 (0)	0.5055 (0)
BGLR	Wheat_1_AL	0.0489 (0)	0.5052 (0)
glmnet	Wheat_2_AL	0.0454 (0)	0.1167 (0)
glmnet-M	Wheat_2_AL	0.0431 (0)	0.3242 (0)
rrBLUP	Wheat_2_AL	0.0419 (0)	0.3948 (0)
BGLR	Wheat_2_AL	0.0419 (0)	0.3937 (0)
glmnet	Wheat_3_AL	0.0452 (0)	0.3268 (0)
glmnet-M	Wheat_3_AL	0.0416 (0)	0.4696 (0)
rrBLUP	Wheat_3_AL	0.0414 (0)	0.4781 (0)
BGLR	Wheat_3_AL	0.0414 (0)	0.4773 (0)
glmnet	Wheat_4_AL	0.0462 (0)	0.1334 (0)
glmnet-M	Wheat_4_AL	0.0438 (0)	0.3349 (0)
rrBLUP	Wheat_4_AL	0.0431 (0)	0.3775 (0)
BGLR	Wheat_4_AL	0.0431 (0)	0.3761 (0)
glmnet	Wheat_5_AL	0.0404 (0)	0.321 (0)
glmnet-M	Wheat_5_AL	0.038 (0)	0.4318 (0)
rrBLUP	Wheat_5_AL	0.0376 (0)	0.4486 (0)
BGLR	Wheat_5_AL	0.0377 (0)	0.4475 (0)
glmnet	Wheat_6_AL	0.0461 (0)	0.3528 (0)
glmnet-M	Wheat_6_AL	0.041 (0)	0.5253 (0)
rrBLUP	Wheat_6_AL	0.0403 (0)	0.548 (0)
BGLR	Wheat_6_AL	0.0403 (0)	0.5476 (0)

SD represents the standard deviation across traits and across folds, with glmnet and glmnet-M methods in each dataset (Dataset). Note that because there is only one trait in the Maize and Wheat_1-Wheat_6 datasets, the corresponding SD is 0.

**Table 3.** Average NRMSE and average Cor across different datasets (dataset) of the mean values obtained across traits, with glmnet and glmnet-M methods.

Method	NRMSE (SD)	Cor (SD)
glmnet	9.0614 (33.509)	0.2871 (0.1053)
glmnet-M	9.0538 (33.5128)	0.4483 (0.1131)
rrBLUP	8.9777 (33.2317)	0.473 (0.1052)
BGLR	8.9794 (33.2381)	0.473 (0.1051)

SD represents the standard deviation of the metric across different datasets of the mean values obtained across traits.

### Disease

In the Disease dataset, Fig. 1 displays the comparative evaluation results between glmnet and glmnet-M approaches (Tables 1 and 2), revealing subtle yet significant differences in their predictive performance. In terms of NRMSE, glmnet-M consistently shows a marginal improvement over glmnet for the PTR and SB traits, with average differences of 0.0035 and 0.0058, respectively. Regarding Cor, glmnet-M displays a notably superior performance for the same traits, outperforming glmnet with average differences of 0.092 and 0.068 respectively, both also with 95% CI, indicating a significant difference. However, in the SN trait, while glmnet shows a slightly lower NRMSE than glmnet-M, the difference is not statistically significant (average difference of 0.0017 with a confidence interval including 0), although glmnet-M exhibits a marginally higher Pearson Correlation.

Consequently, we concluded that glmnet-M prevails as the preferred model in most of the traits evaluated, due to its consistent improvement in predictive accuracy, as evidenced by the NRMSE and Pearson Correlation. Overall, glmnet-M is 0.59% better than glmnet for the NRMSE metric, while for COR, glmnet-M is 34.16% better.

### EYT_1

In Fig. 2, the results of the glmnet and glmnet-M methods in the “EYT_1” dataset (Tables 1 and 2) suggest substantial differences in their predictive capacity for the 4 traits (DTHD, DTMT, GY, and Height). Across all the evaluated traits, glmnet-M consistently displays a significantly higher performance compared with glmnet, as evidenced by both the NRMSE and Cor. In terms of NRMSE, glmnet-M presents a significant improvement across all traits, with average differences of 0.0048, 0.0042, 0.0056, and 0.0028 for DTHD, DTMT, GY, and Height, respectively. Similarly, Cor shows a substantial improvement for glmnet-M across all traits, with average differences of 0.2095, 0.2076, 0.1893, and 0.1991 for DTHD, DTMT, GY, and Height, respectively.

Therefore, we concluded that glmnet-M prevails as the preferred method for all the evaluated traits in the “EYT_1” dataset, given its statistically greater performance in terms of predictive error and correlation. Overall, glmnet-M is 11.33% better than glmnet for the NRMSE metric, whereas for Cor, glmnet-M is 76.15% better.

### Indica

For the “Indica” dataset and the trait “GC,” the glmnet-M model outperforms the glmnet model in terms of NRMSE (Tables 1 and 2), with an average value of 0.4439 compared with glmnet's 0.4568. This represents an improvement of approximately 3.02% (Fig. 3). Additionally, in terms of Cor, the glmnet-M model also surpasses the glmnet method, with an average value of 0.3299 compared with glmnet-M 0.2396. This represents an improvement of approximately 37.76%. In summary, the glmnet-M model is superior to the glmnet model for the “GC” trait in the “Indica” dataset, with significant improvements in both evaluation metrics.

For the GY trait, the glmnet-M has an average NRMSE of 0.0549, whereas the glmnet has an average NRMSE of 0.063. This represents a 12.96% improvement in the NRMSE of the glmnet-M method against the glmnet. Regarding Cor, the glmnet-M model has an average value of 0.6392, which is higher than the glmnet method, with an average value of 0.636.

For the PH trait, the glmnet-M has an average NRMSE of 0.0425, whereas the glmnet has an average NRMSE of 0.0451. This represents a 5.76% improvement in the NRMSE of the glmnet-M method regarding the conventional approach (glmnet). Additionally, in terms of Cor, the glmnet-M model has an average value of 0.5334, which is higher than the glmnet method, with an average value of 0.5447.

For the PHR trait, the glmnet-M has an average NRMSE of 0.0349, whereas the glmnet has an average NRMSE of 0.0369. This represents a 5.42% improvement in the NRMSE. Regarding Cor, the glmnet-M has an average value of 0.3537, which is higher than the glmnet average value of 0.2493. Overall, glmnet-M is better by 4.44% compared with glmnet for the NRMSE metric, while for Cor, glmnet-M is 11.16% better.

### Wheat_1-Wheat_6

In the “Wheat_1” dataset, the glmnet-M method performed better, as it has an average NRMSE of 0.0502 and an average Pearson Correlation of 0.4648, whereas the glmnet has an average NRMSE of 0.0555 and an average Pearson Correlation of 0.2262. Consequently, glmnet-M is superior by 10.56% compared with glmnet in terms of the NRMSE metric, while in terms of Cor, glmnet-M is superior by 105.48% (Fig. 4, Tables 1 and 2).

In the “Wheat_2”, “Wheat_3”, “Wheat_4”, “Wheat_5”, and “Wheat_6” datasets, a better performance of glmnet-M was observed compared with glmnet, expressed as a lower average NRMSE and a higher average Pearson Correlation in all datasets. This suggests that the glmnet-M model is more effective for these specific datasets in terms of prediction error (NRMSE) and Cor. Overall, glmnet-M is better by an interval of 5.34 to 12.44% compared with glmnet for the NRMSE metric, while for Pearson Correlation, glmnet-M is between 34.52 and 177.81% better.

### Across trait

In Fig. 5, the average NRMSE and average Pearson Correlation are presented for each dataset, comparing the glmnet and glmnet-M methods. The average NRMSE value for glmnet is 9.0614, with a SD of 33.509, whereas for glmnet-M, it is slightly lower, with a value of 9.0538 and a SD of 33.5128. Regarding the average Pearson Correlation, glmnet-M has a considerably higher value, with an average of 0.4483 and a SD of 0.1131, compared with glmnet, which has an average of 0.2871 and a SD of 0.1053. This suggests that the glmnet-M method tends to produce more accurate predictions across a variety of datasets compared with glmnet. Since in terms of Cor the average gain of the proposed method glmnet-M was 56.15% regarding the conventional method (glmnet), no significant differences were observed in terms of NRMSE.

Table 3 gives the average NRMSE and average Cor across different datasets (Dataset) of the mean values obtained across traits, with glmnet and glmnet-M methods, Overall results show an important increase in correlation between observed and predicted values when using the glment-M (0.4483) over the standard glment (0.2871).

**Fig. 6.**
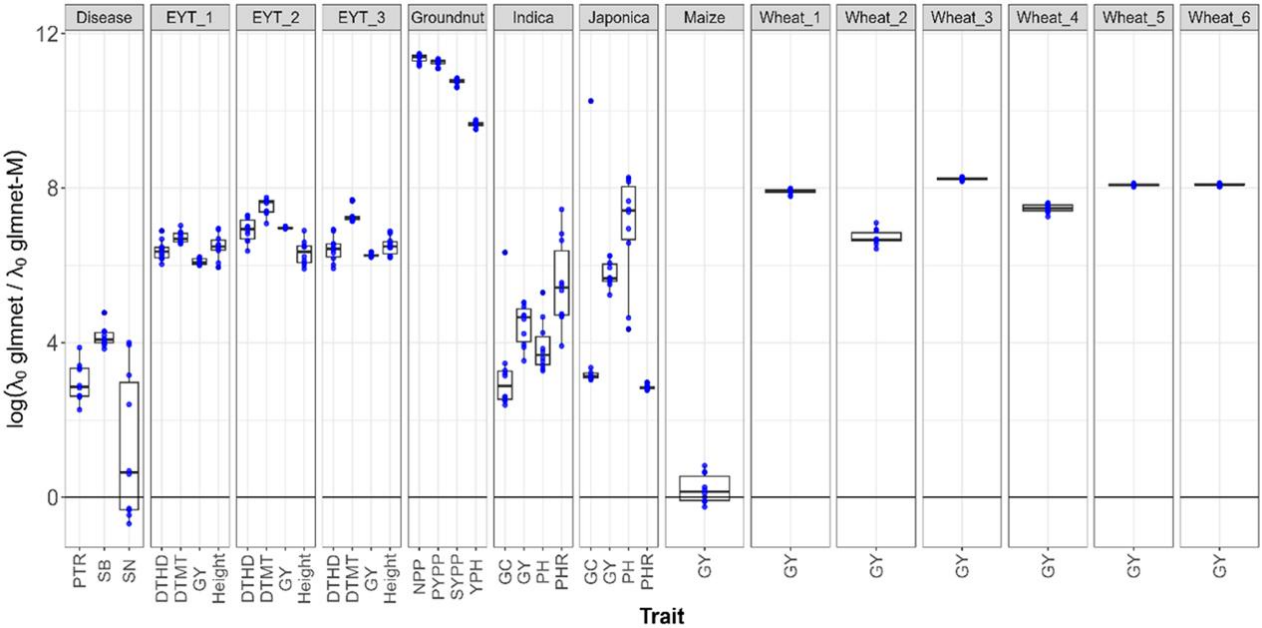
Boxplots of the logarithm of the ratio of optimal lambda values for the glmnet and glmnet-M methods obtained in each fold during the 10-fold cross-validation for each trait and each dataset.

**Fig. 7.**
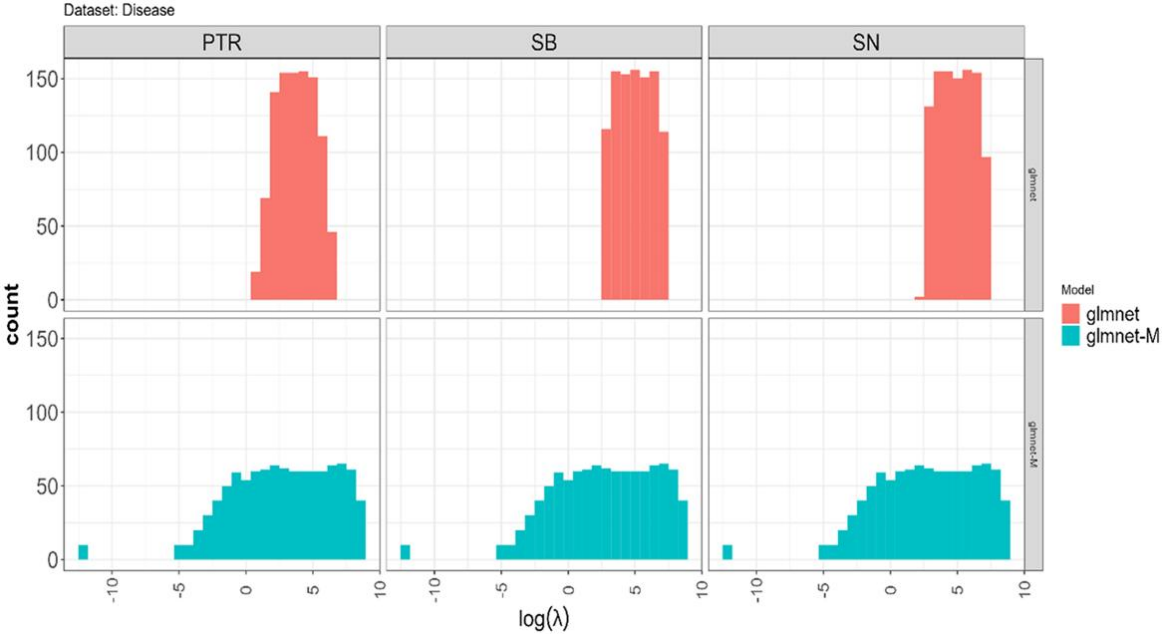
Histograms of the log(lambda) grid values in the glmnet and glmnet-M methods across all folds in the 10-fold cross-validation.

Furthermore, boxplots of the logarithm of the ratio of the “optimal” lambda values ($\log\;(\lambda_{O\mathrm{glmnet}}\;/\lambda_{O\mathrm{glmnet}-M\;})$) found in the tunning process with the glmnet ($\lambda_{O\mathrm{glmnet}}$) and glmnet-M ($\lambda_{O\mathrm{glmnet}-M\;}$) methods, obtained in each fold during the 10-fold cross-validation evaluation, are shown in Fig. 6. From this, we can observe that for all datasets, except for the traits SN (Disease_AL data) and GY (Maize data), in which only 6 out of the 10 folds and 7 out of 10 folds, respectively, this $\log\;(\lambda_{O\mathrm{glmnet}}\;/\lambda_{O\mathrm{glmnet}-M\;})$ values are > 0. This indicates that the ratio of the “optimal”, $\lambda_{O\mathrm{glmnet}}\;/\lambda_{O\mathrm{glmnet}-M}$, are larger than 1 and therefore, the penalization strength in the glmnet method tends to be much higher than in glmnet-M. This is a consequence of the larger search space of the grid lambda values used in the tunning process with the glmnet-M method compared with the corresponding grid used in the glmnet method. As shown in Fig. 7 for the Disease data, the support of the histogram of logarithmic lambda grid values in the glmnet method is entirely contained within the support of the histogram for the glmnet-M method, that is, the range of the histogram of the logarithmic lambda grid values in the glmnet method is entirely within the range of the histogram of lambda grid values in the glmnet-M method. Although not shown, a very similar behavior was observed in the other datasets.

Finally, in the same figures the results of the performance evaluation of the glmnet and glmnet-M methods were reported, Figs. 1–4 and Figs. B1–B5 (Appendix B), the corresponding performance predictions of rrBLUP (Endelman 2011) and BGLR (Pérez and de Los Campos 2014) were also included for comparative purposes. These correspond to the classic GBLUP and Bayesian GBLUP prediction methods, respectively. These are 2 of the current workhorses in genomic prediction due to their powerful prediction performance. We can observe that in all datasets, the proposed modified tuning parameter glmnet method, glmnet-M, is very competitive with respect to these 2 additional methods (rrBLUP and BGLR) in both evaluated metrics, except in the Wheat_2 and Wheat_4 datasets, where our proposal shows a slightly less competitive performance according to the Pearson correlation metric.

## Discussion

Ridge regression continues to be a popular statistical learning algorithm for genomic prediction, mainly due to its accuracy, simplicity, and the availability of user-friendly software. For these reasons, it has been implemented in diverse fields such as finance, economics, medicine and healthcare, marketing, geophysics and geology, engineering, social sciences, image and signal processing, text mining, and natural language processing, among others.

Although Ridge regression is widely used, it is important to highlight that, in the version analyzed in this paper, the method assumes a linear relationship between the features and the target variable. When this assumption is not met, the model's predictive performance may be compromised, as Ridge regression is unable to capture nonlinear patterns in the data. It is important to note that Ridge regression is not limited to modeling linear relationships; it can also efficiently handle nonlinear patterns. Additionally, the prediction accuracy of this method depends on the bias-variance tradeoff. Ridge regression introduces bias to reduce variance, which can sometimes lead to underfitting, especially if the regularization parameter (*λ*) is too high. Therefore, the optimal selection of *λ* is critical to balance model complexity and generalization performance. It controls the tradeoff between overfitting and underfitting, ensuring the model captures relevant patterns in the data without memorizing noise. By tuning *λ*, practitioners can improve the model's interpretability by adjusting the magnitude of coefficients. Additionally, *λ* helps stabilize coefficient estimation in the presence of multicollinearity, enhancing the model's robustness. Ultimately, selecting the right *λ* maximizes the model's predictive accuracy and reliability on unseen data.

To guarantee the best Ridge regression performance, the optimal selection of the regularization parameter *λ* is essential. The prevailing method to determine the optimal regularization parameter involves setting a range of *λ* values and a grid resolution for grid search, as outlined in the materials and methods section. However, the chosen grid resolution can significantly influence the selected *λ* value, potentially leading to suboptimal outcomes if the true optimal value is not covered by the grid points. Moreover, the prevalent methods to select the optimal *λ* through cross-validation and grid search primarily focus on optimizing model performance, often neglecting a thorough understanding of the relationships between predictors and the target variable. Consequently, the chosen *λ* value may lack interpretability and a robust connection with the target variable, increasing the likelihood of not selecting the truly optimal *λ*.

To improve the optimal selection of the regularization parameter (*λ*), we propose a method that selects the grid of *λ* values by computing each component of *λ* as a proportion of the phenotypic response in the training set. This method is inspired by how priors are given in Bayesian ridge regression (details are provided in Appendix 2, Chapter 6 of the book by Montesinos-López *et al*. 2022). Our results, evaluated on 14 real datasets, show significant gains—around 56.15% improvement in terms of Cor—although no gains were observed in terms of NRMSE. The observed gain can be attributed to the efficiency of the proposed method to select *λ* values for the grid that are strongly related to the inputs and the target variable. Thus, the proposed method enhances existing alternatives to select the optimal regularization parameter (*λ*).

In general, the proposed approach for the optimal selection of the regularization parameter (*λ*) in Ridge regression is of paramount importance as it enhances the prediction power of one of the most popular linear models used in many areas of science. Ridge regression is particularly important in genomic prediction due to its ability to handle multicollinearity among genetic markers, providing more stable and reliable estimates. It effectively shrinks coefficients, reducing overfitting and enhancing model generalizability. Ridge regression also accommodates large-scale genomic data by penalizing the magnitude of regression coefficients, thereby managing the high dimensionality characteristic of genomic datasets.

Additionally, it aids in the inclusion of all available markers, which is crucial for capturing the complex genetic architecture of traits. Ultimately, its regularization properties improve the predictive accuracy and robustness of genomic studies. Furthermore, the Supplementary Materials demonstrate that the proposed method is effective not only for Ridge regression but also to tune the regularization parameter (*λ*) in Lasso (*α*=1) and Elastic Net (0<*α*<1) regression. The most significant improvement was observed in Ridge regression, with the least improvement in Lasso regression. According to the notation used in the glmnet library, Ridge regression is implemented when *α*=0, Lasso regression when *α*=1, and Elastic Net regression when 0<*α*<1. Therefore, the proposed method is highly attractive to enhance prediction performance in penalized regression models.

Additionally, we acknowledge that the proposed method can be extended to other types of response variables within the context of Ridge regression. With relatively straightforward modifications, it can be generalized to penalized binomial, Poisson, and other types of response variables in penalized regression model. Also, in future works, the proposed method can be compared with the method of Pavlou *et al*. (2024) proposed in the context of logistic regression for binary response variables.

### Conclusions

In this article, we propose a more efficient approach for selecting the regularization parameter for Ridge regression. Using 14 datasets, we show that the proposed method outperformed the conventional method in 13 of them. The gains obtained were 56.09% in terms of Cor, with no significant differences observed in terms of the NRMSE across the 14 datasets. Therefore, we encourage the use of the proposed method to increase empirical evidence of its ability to enhance the prediction performance of Ridge regression. Although there are many statistical machine learning methods currently used for genomic prediction, the improvements obtained with our method to efficiently tune the regularization parameter of the Ridge regression may help it remain one of the most popular algorithms in the context of genomic prediction.

## Supplementary Material

jkae246_Supplementary_Data

## Data Availability

The data and code used in this publication are available at: https://github.com/osval78/Refaning_Penalized_Regression. The Supplementary Material contains Figs. 1–20 and Tables 1–3. Supplemental material available at G3 online.

## Appendix A

**Table A1.** Brief data description.

Data	No. Lines	No. Markers	Multi-Environment data	BLUEs across environments	Experimental design
Indica	327	16,383	YES	YES	RCBD
Japonica	320	16,383	YES	YES	RCBD
Groundnut	318	8,268	YES	YES	Alpha-lattice
Maize	722	54,113	YES	YES	RCBD
Wheat_1	1,301	78,606	YES	YES	Alpha-lattice
Wheat_2	1,403	78,606	YES	YES	Alpha-lattice
Wheat_3	1,403	78,606	YES	YES	Alpha-lattice
Wheat_4	1,388	78,606	YES	YES	Alpha-lattice
Wheat_5	1,398	78,606	YES	YES	Alpha-lattice
Wheat_6	1,277	78,606	YES	YES	Alpha-lattice
EYT_1	776	2,038	YES	YES	Alpha-lattice
EYT_2	775	2,038	YES	YES	Alpha-lattice
EYT_3	964	2,038	YES	YES	Alpha-lattice
Disease	438	11,617	YES	YES	RCBD

RCBD denotes randomized complete block design, while alpha-lattice denotes the alpha lattice experimental design.

### Setting each component of lambda $\lambda =\sigma^{2}/\sigma_{\beta }^{2}$ as a proportion of the total response variable

In Appendix 2 of Chapter 6 of Montesinos-López *et al*. (2022) express the total variance explained by the response variable of model (1) in terms of the inputs (markers) and error component as:

(A1 )
$$\mathrm{Var}(y_{j})=\mathrm{Var}(x_{j}^{T}\beta )+\sigma^{2}$$

Therefore, the average of the variance of the individuals, called total variance, is equal to

$$\begin{array}{rl} \frac{1}{n_{\mathrm{trn}}}\overset{n_{\mathrm{trn}}}{\underset{\;j=1}{\sum }}\;\mathrm{Var}(y_{j}) & =\frac{1}{n_{\mathrm{trn}}}\overset{n_{\mathrm{trn}}}{\underset{\;j=1}{\sum }}\;\mathrm{Var}(x_{j}^{T}\beta )+\sigma^{2}=\frac{1}{n_{\mathrm{trn}}}\mathrm{tr}(XX^{T})\sigma_{\beta }^{2}+\sigma^{2}=V_{M}\\ & \;+V_{\epsilon } \end{array}.$$

Then, by setting $R_{1}^{2}$ as a proportion of the total variance ($s_{y}^{2}$), that is explained by inputs a priori, $V_{M}=R_{1}^{2}s_{y}^{2}$, and equating $\frac{1}{n_{\mathrm{trn}}}\mathrm{tr}(XX^{T})\sigma_{\beta }^{2}$ to $V_{M}=R_{1}^{2}s_{y}^{2}$

$$\frac{1}{n_{\mathrm{trn}}}\mathrm{tr}(XX^{T})\sigma_{\beta }^{2}=R_{1}^{2}s_{y}^{2}.$$

From here, once we rewrite the variance for $\sigma_{\beta }^{2}$ as

$$\sigma_{\beta }^{2}=\frac{R_{1}^{2}s_{y}^{2}}{\frac{1}{n_{\mathrm{trn}}}\mathrm{tr}(XX^{T})}=\frac{R_{1}^{2}s_{y}^{2}}{\frac{1}{n_{\mathrm{trn}}}\sum_{i=1}^{n_{\mathrm{trn}}}\;x_{i}^{T}x_{i}}$$

Since the expression given in Equation (A1) only has 2 components and $R_{1}^{2}$ was set as the proportion of the total variance that is explained by inputs a priori, the corresponding proportion that is explained by error a priori is $R_{2}^{2}=1-R_{1}^{2}$. Therefore, the variance of the error component expressed as a proportion of variance of the total variance can be expressed as

$$\sigma^{2}=(1-R_{1}^{2})s_{y}^{2}\;$$

For this reason, *λ* can be expressed as:

$$\lambda =\frac{\sigma^{2}}{\sigma_{\beta }^{2}}=\frac{(1-R_{1}^{2})s_{y}^{2}}{R_{1}^{2}s_{y}^{2}/\left(\frac{1}{n_{\mathrm{trn}}}\sum_{i=1}^{n_{\mathrm{trn}}}\;x_{i}^{T}x_{i}\right)}=\frac{1-R_{1}^{2}}{R_{1}^{2}/\left(\frac{1}{n_{\mathrm{trn}}}\sum_{i=1}^{n_{\mathrm{trn}}}\;x_{i}^{T}x_{i}\right)}$$

## Appendix B

Figures for datasets EYT_2 (Figure B1), EYT_3 (Figure B2), Groundnut (Figure B3), Japonica (Figure B4), and maize (Figure B5).
