# Supplementary material for: Refining penalized Ridge regression: a novel method for optimizing the regularization parameter in genomic prediction
Source: G3 (Bethesda). 2024 Nov 9;14(12):jkae246. doi: 10.1093/g3journal/jkae246 (PMC11631504; doi:10.1093/g3journal/jkae246)
Supplement: jkae246_Supplementary_Data [file jkae246_supplementary_data.docx]

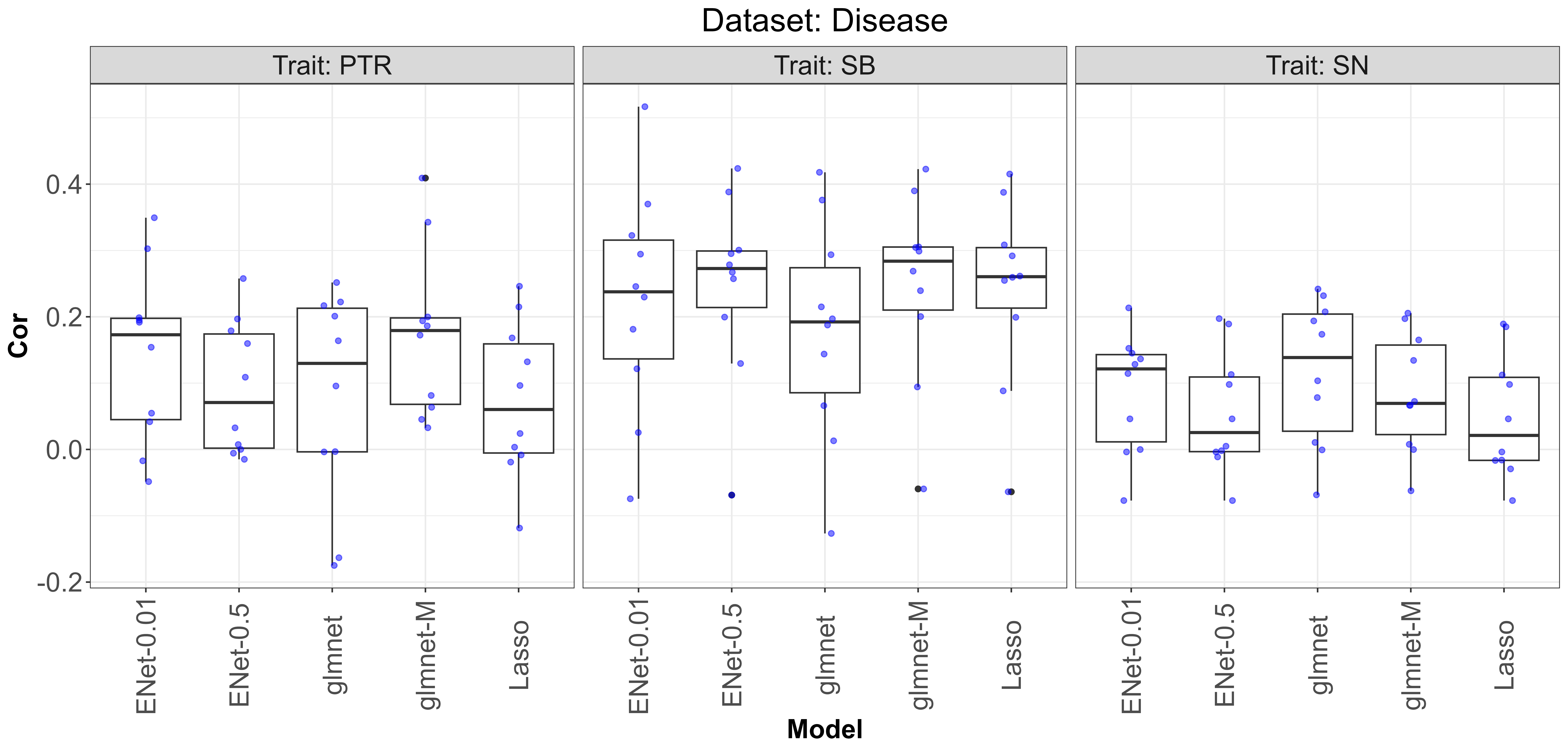


**Figure S1**. Box Plot of prediction performance in terms of Pearson’s Correlation (Cor) between observed and predicted values through ten-fold cross-validation for each of the three traits (PTR, SB, and SN) of the **Disease** dataset with Elastic net with $\alpha=0.01 \mathrm{and} 0.5$(Enet-0.01; Enet-0.5), Ridge regression (glmnet), theproposed method (glmnet-M) and the Lasso method.


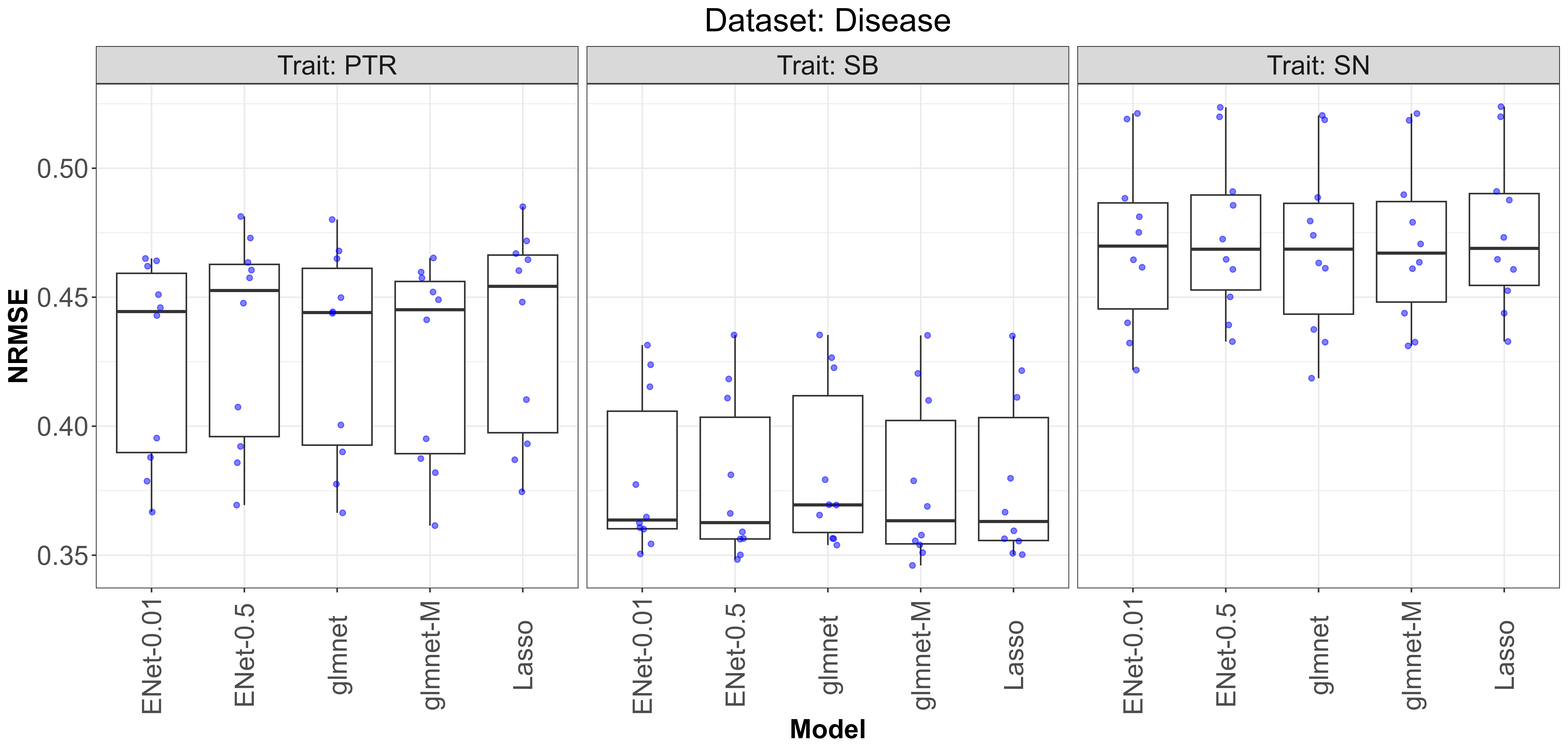


**Figure S*2***. Box Plot prediction performance in terms of the Normalized Root Mean Squared Error (NRMSE) between observed and predicted values through ten-fold cross-validation for each of the three traits (PTR, SB, and SN) of the **Disease** dataset with Elastic net with $\alpha=0.01 \mathrm{and} 0.5$(Enet-0.01; Enet-0.5), Ridge regression (glmnet), the proposed method (glmnet-M) and the Lasso method.


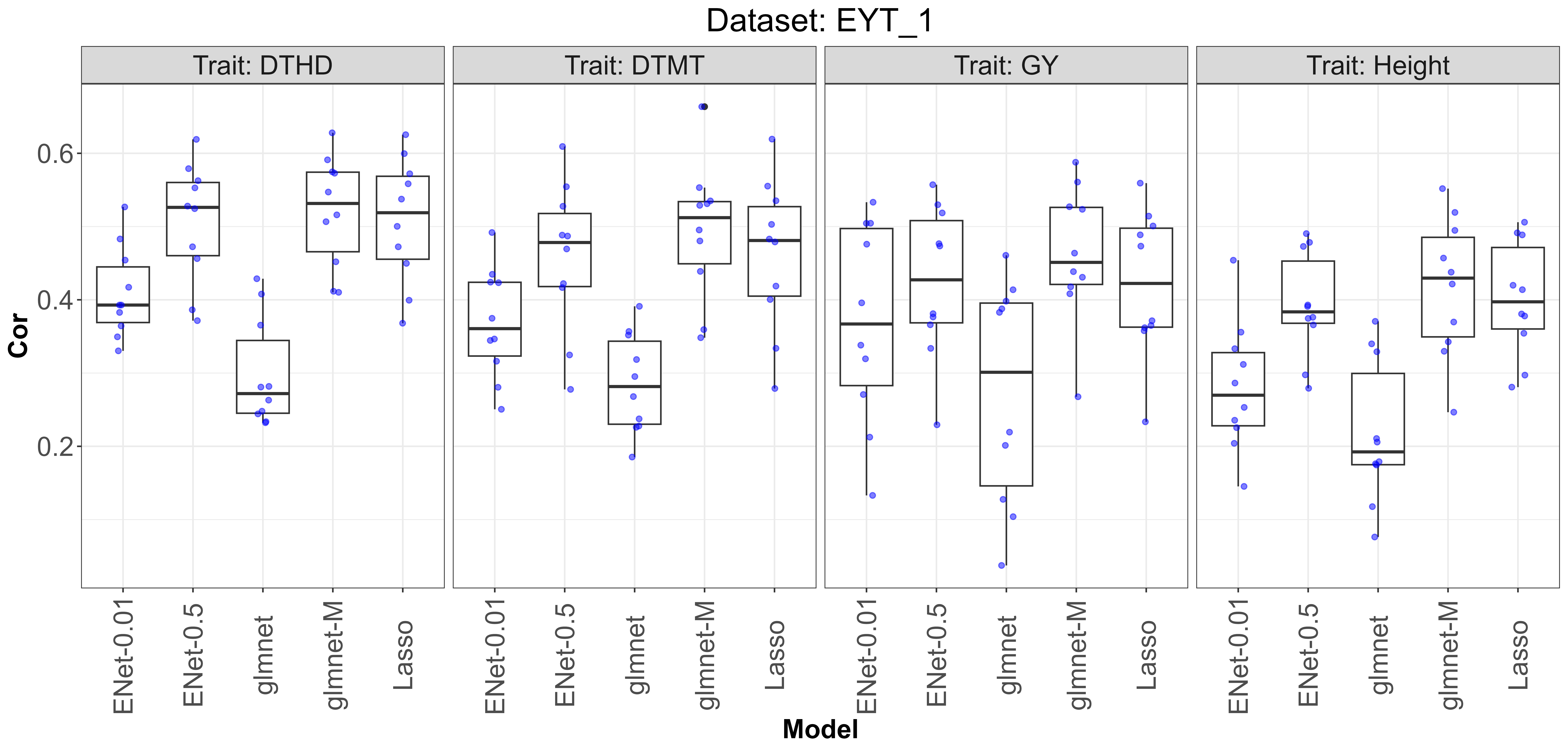


**Figure S3**. Box Plot prediction performance in terms of Pearson’s Correlation (Cor) between observed and predicted values through ten-fold cross-validation for each of the four traits (DTHD, DTMT, GY and Height) of the **EYT_1** dataset with Elastic net with $\alpha=0.01 \mathrm{and} 0.5$(Enet-0.01; Enet-0.5), Ridge regression (glmnet), the proposed method (glmnet-M) and the Lasso method.


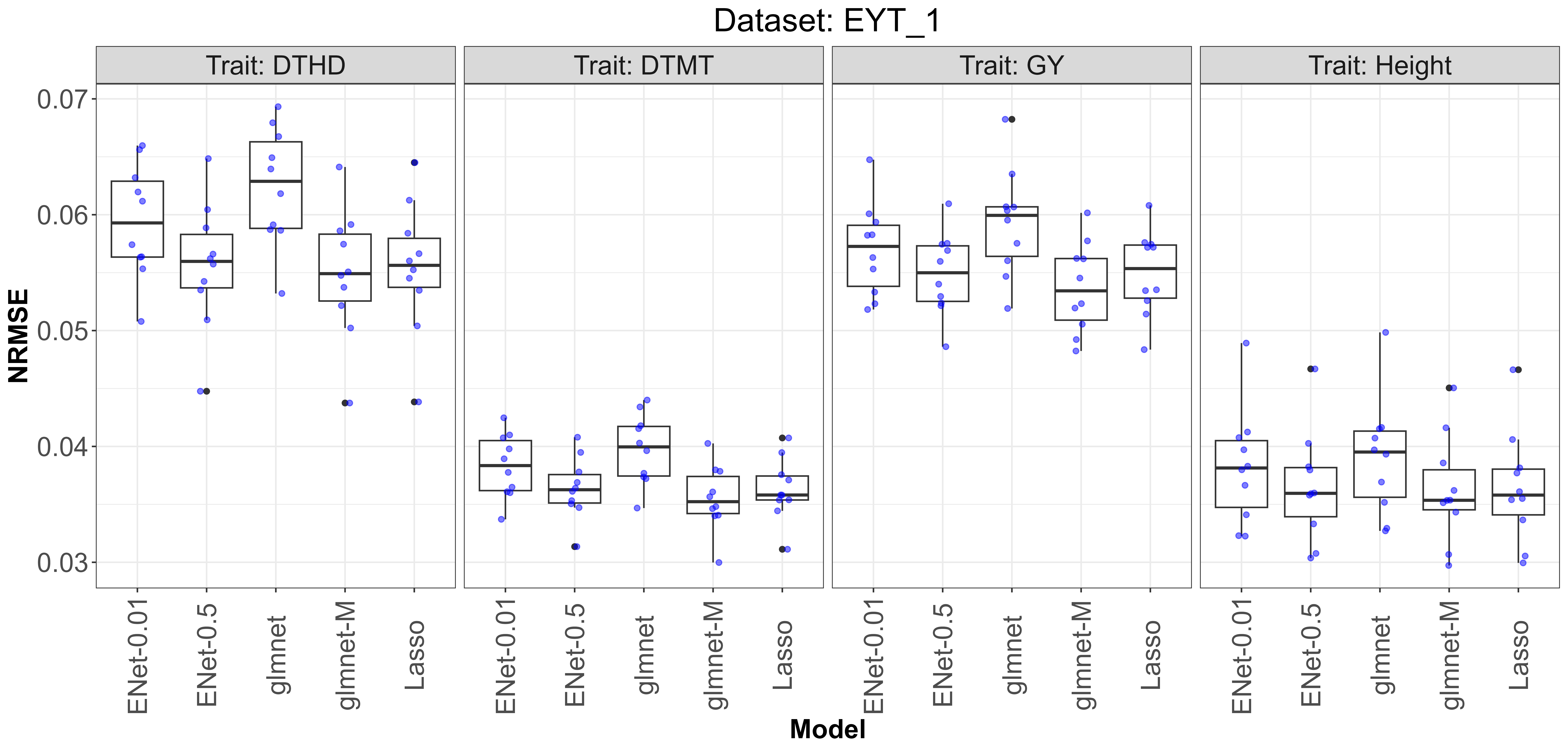


**Figure S*4***. Box Plot prediction performance in terms of the Normalized Root Mean Squared Error (NRMSE) between observed and predicted values through ten-fold cross-validation for each of the four traits (DTHD, DTMT, GY and Height) of the **EYT_1** dataset with Elastic net with $\alpha=0.01 \mathrm{and} 0.5$(Enet-0.01; Enet-0.5), Ridge regression (glmnet), the proposed method (glmnet-M) and the Lasso method.


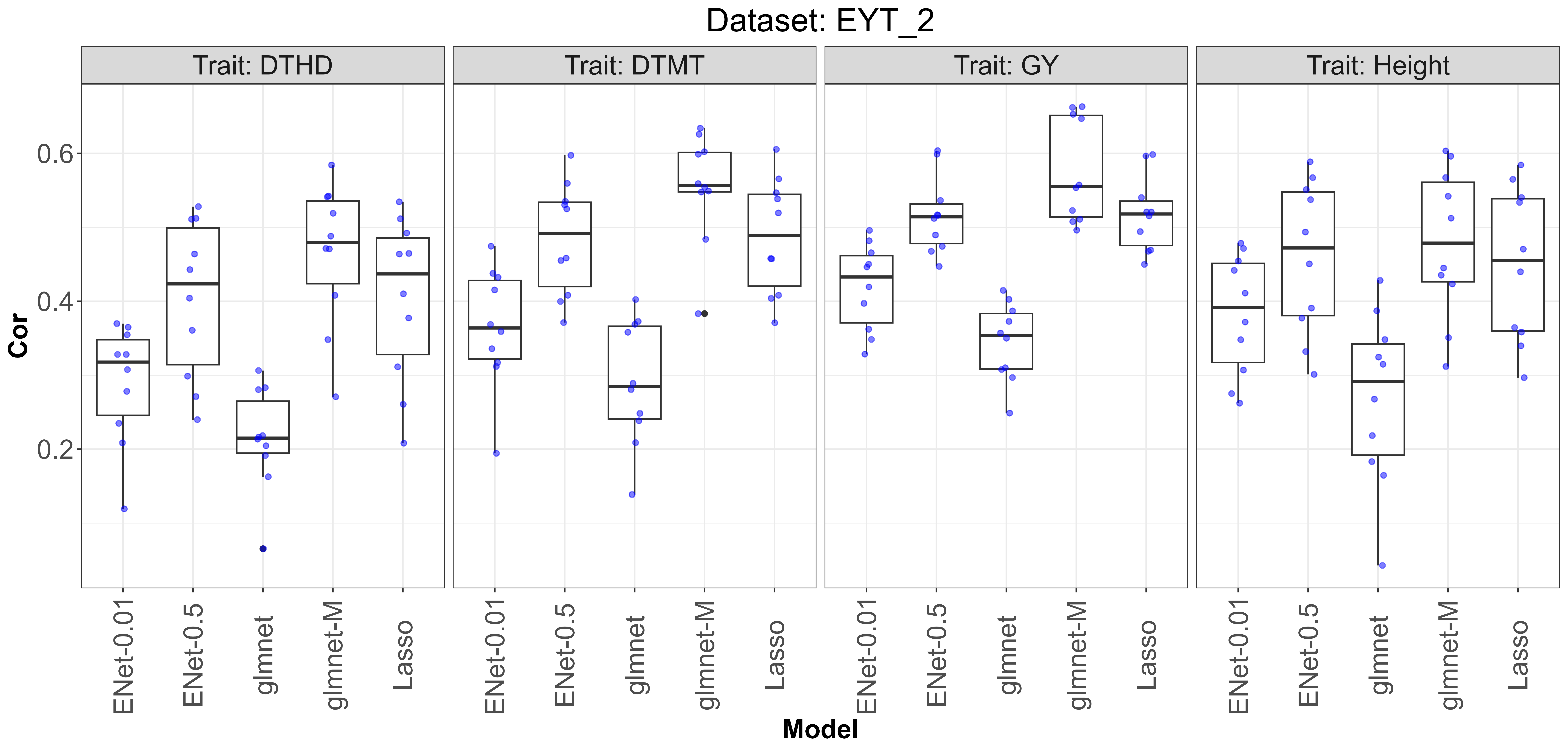


**Figure S5**. Box Plot prediction performance in terms of Pearson’s Correlation (Cor) between observed and predicted values through ten-fold cross-validation for each of the four traits (DTHD, DTMT, GY y Height) of the **EYT_2** dataset with Elastic net with $\alpha=0.01 \mathrm{and} 0.5$(Enet-0.01; Enet-0.5), Ridge regression (glmnet), the proposed method (glmnet-M) and the Lasso method.


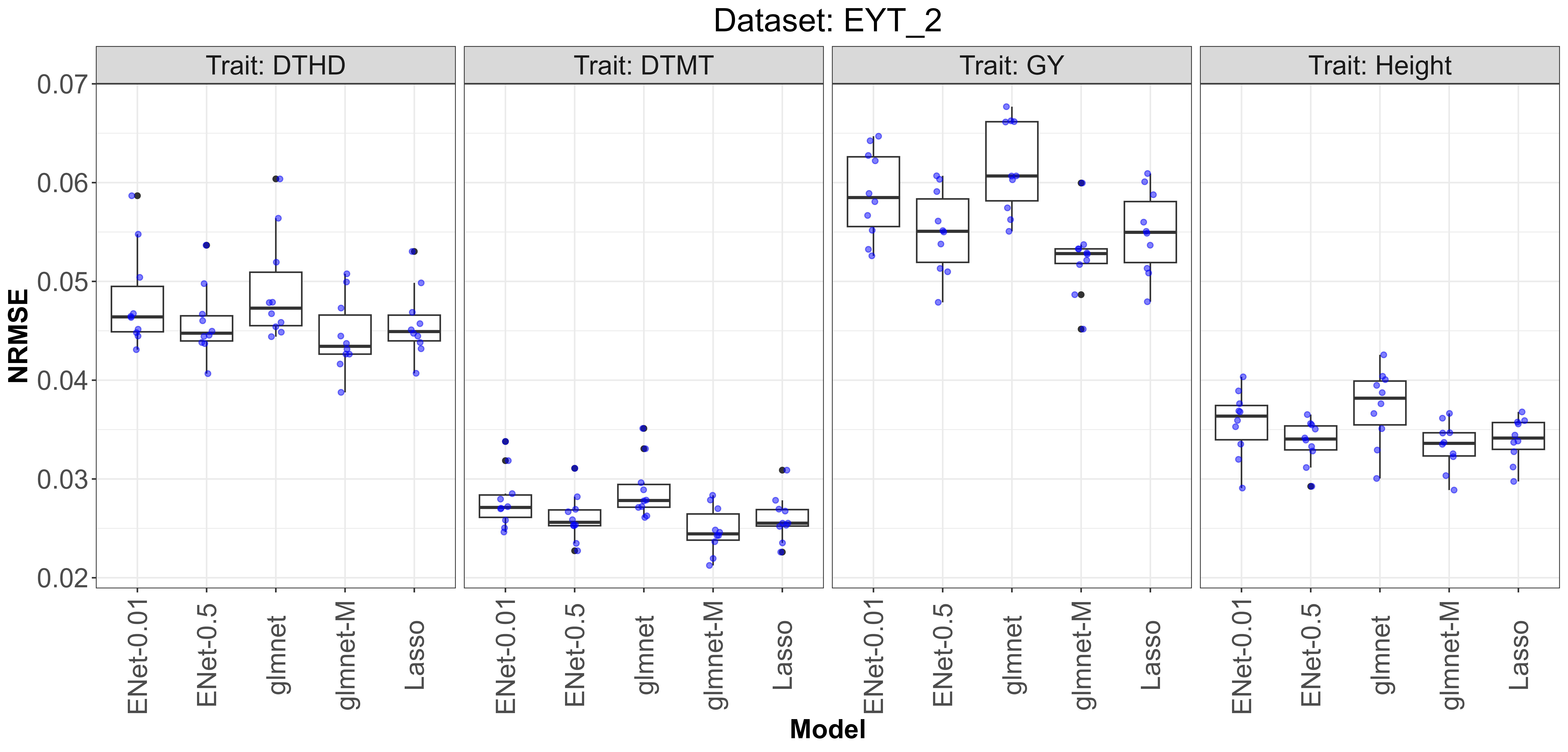


**Figure S*6***. Box Plot prediction performance in terms of the Normalized Root Mean Squared Error (NRMSE) between observed and predicted values through ten-fold cross-validation for each of the four traits (DTHD, DTMT, GY and Height) of the **EYT_2** dataset with Elastic net with $\alpha=0.01 \mathrm{and} 0.5$(Enet-0.01; Enet-0.5), Ridge regression (glmnet), the proposed method (glmnet-M) and the Lasso method.


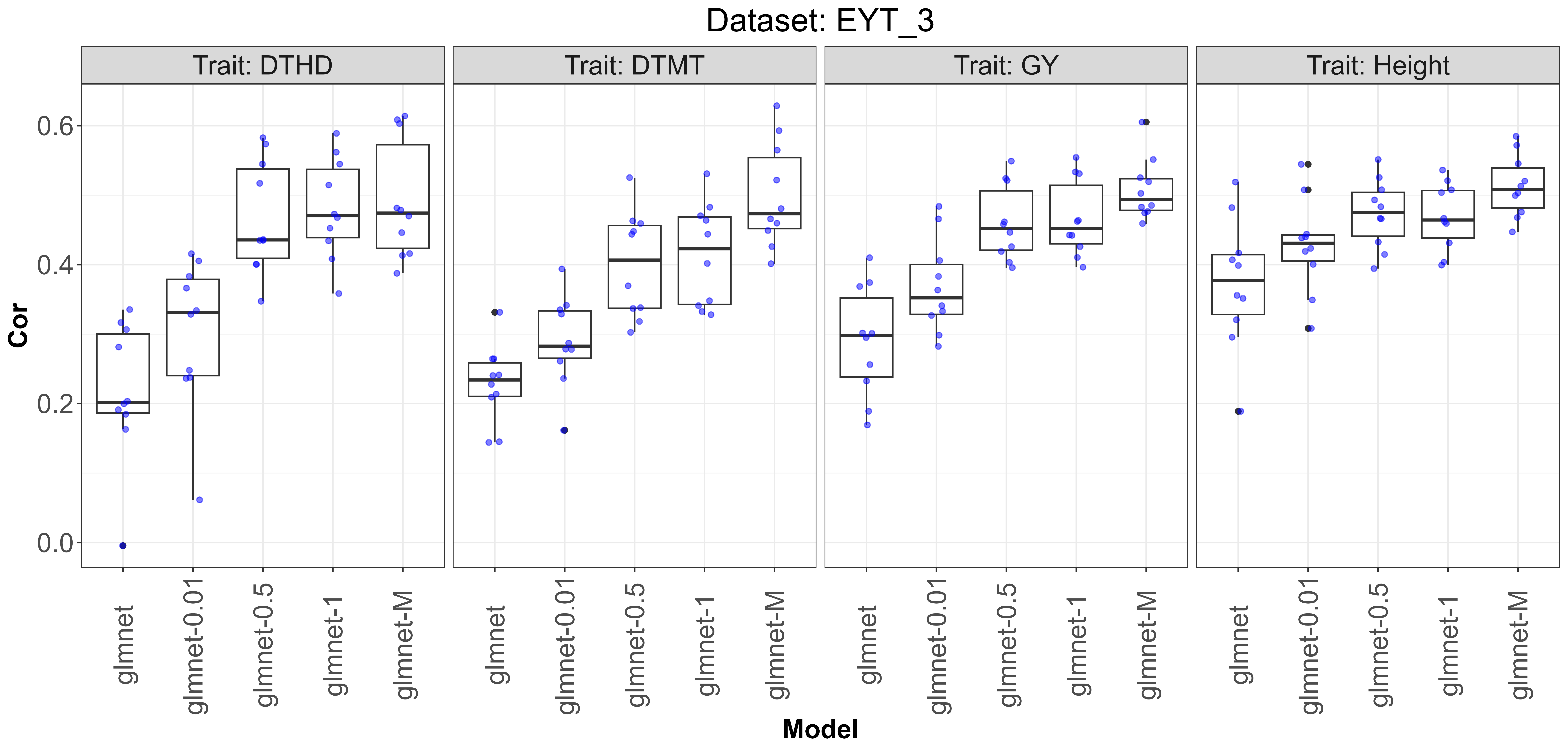


**Figure S7**. Box Plot prediction performance in terms of Pearson’s Correlation (Cor) between observed and predicted values through ten-fold cross-validation for each of the four traits (DTHD, DTMT, GY and Height) of the **EYT_**3 dataset with Elastic net with $\alpha=0.01 \mathrm{and} 0.5$(Enet-0.01; Enet-0.5), Ridge regression (glmnet), the proposed method (glmnet-M) and the Lasso method.


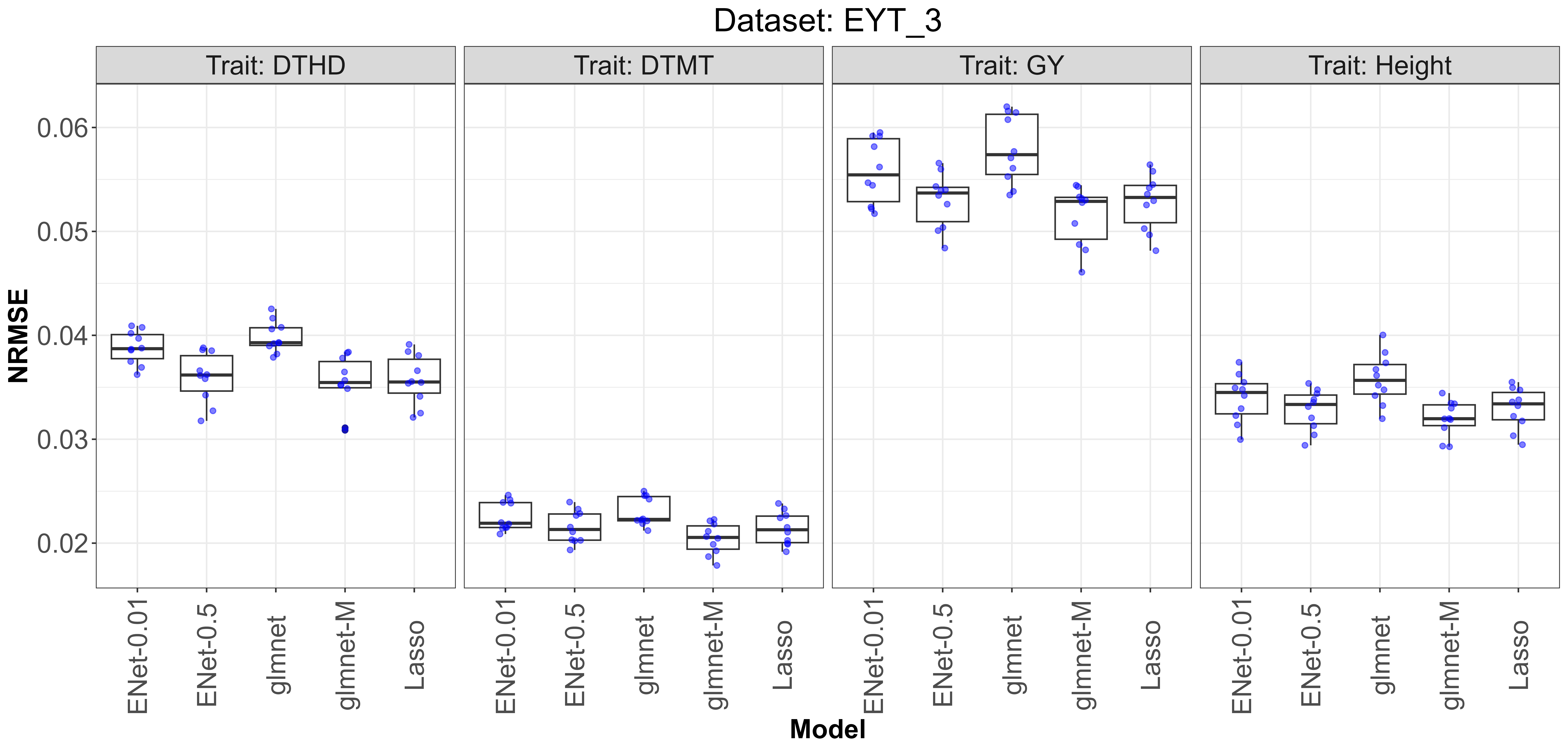


**Figure S*8***. Box Plot prediction performance in terms of the Normalized Root Mean Squared Error (NRMSE) between observed and predicted values through ten-fold cross-validation for each of the four traits (DTHD, DTMT, GY and Height) of the **EYT_3** dataset with Elastic net with $\alpha=0.01 \mathrm{and} 0.5$(Enet-0.01; Enet-0.5), Ridge regression (glmnet), the proposed method (glmnet-M) and the Lasso method.


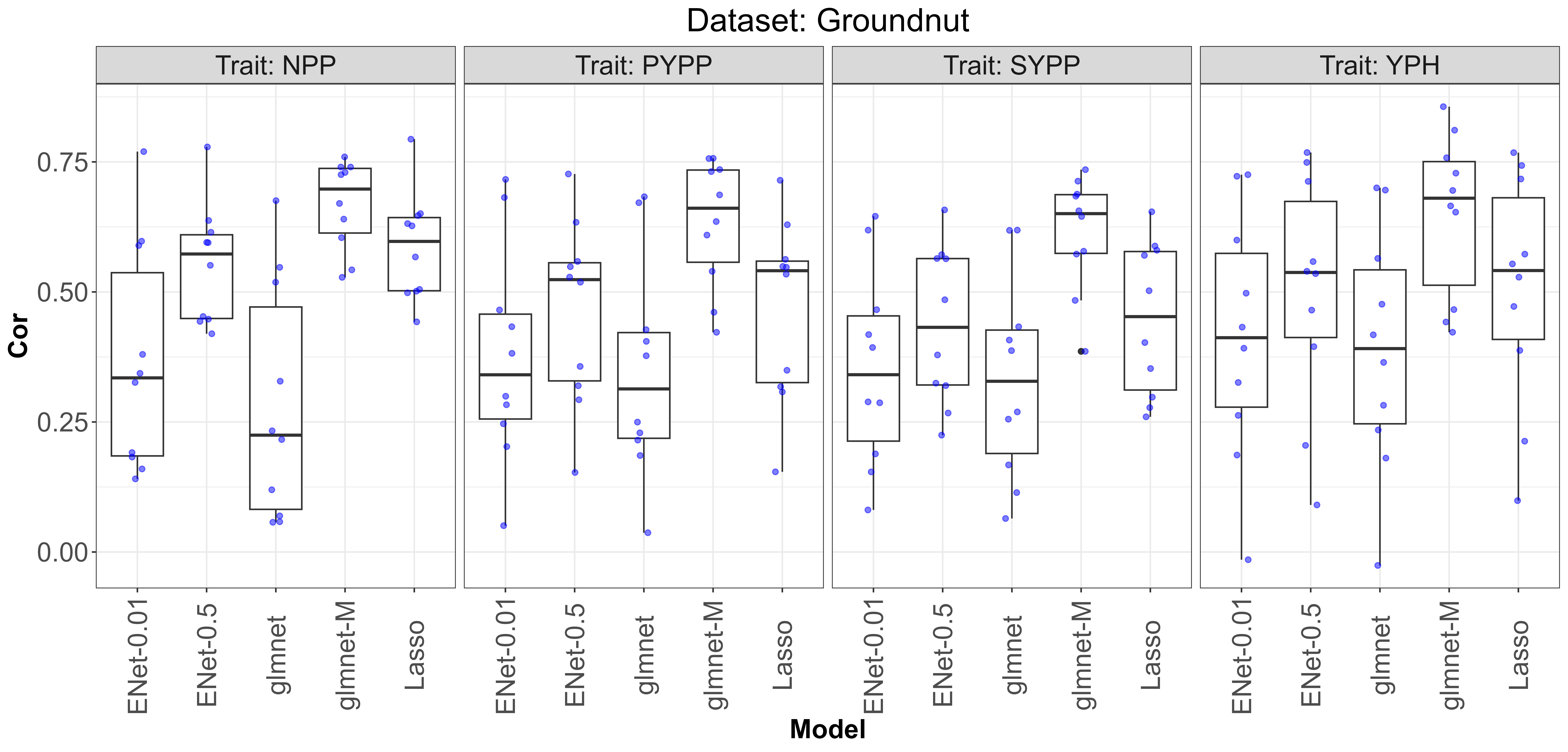


**Figure S9**. Box Plot prediction performance in terms of Pearson's Correlation (Cor) between observed and predicted values through ten-fold cross-validation for each of the four traits (NPP, PYPP, SYPP and YPH) of the **Groundnut** dataset with Elastic net with $\alpha=0.01 \mathrm{and} 0.5$(Enet-0.01; Enet-0.5), Ridge regression (glmnet), the proposed method (glmnet-M) and the Lasso method.


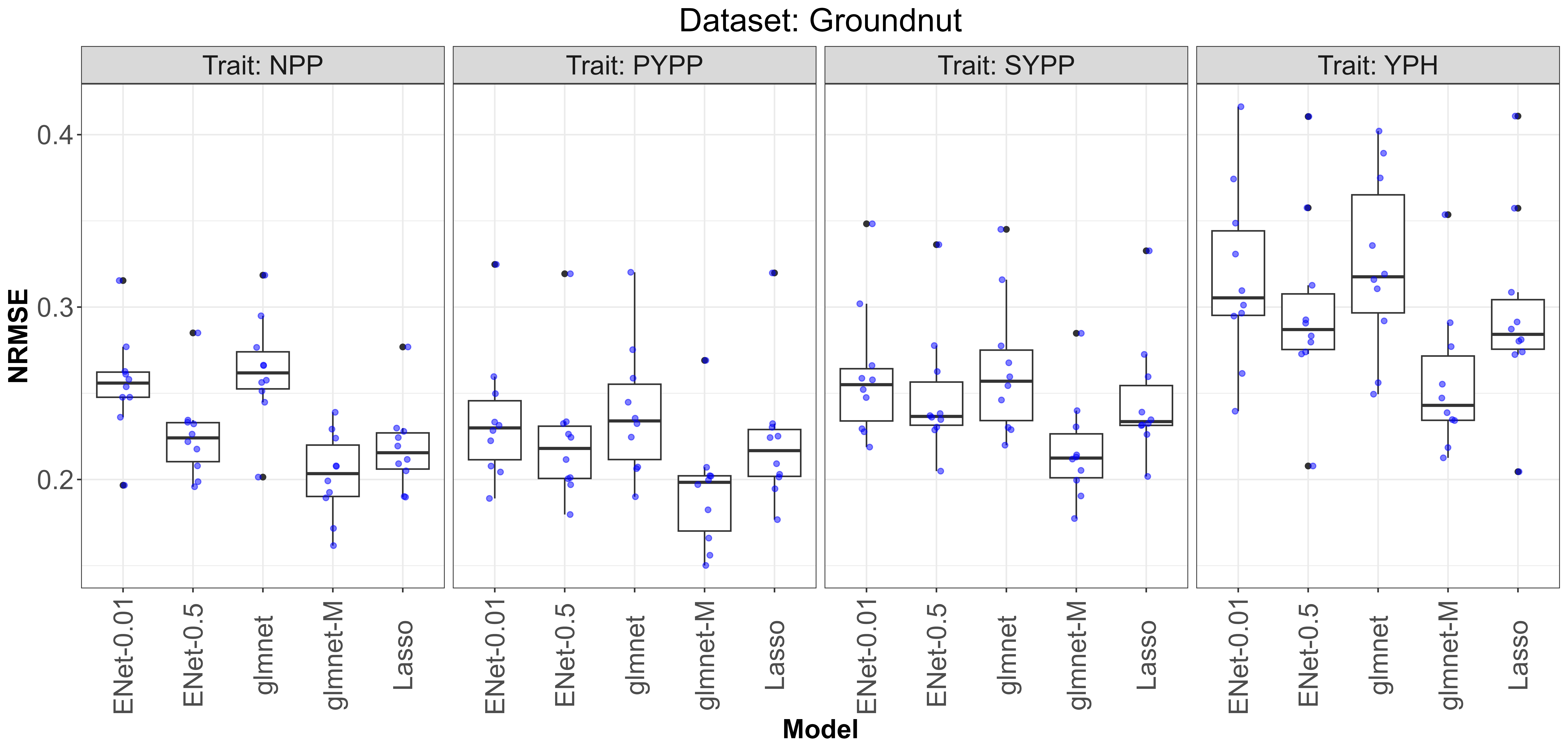


**Figure S*10***. Box Plot prediction performance in terms of the Normalized Root Mean Squared Error (NRMSE) between observed and predicted values through ten-fold cross-validation for each of the four traits (NPP, PYPP, SYPP and YPH) of the **Groundnut** dataset with Elastic net with $\alpha=0.01 \mathrm{and} 0.5$(Enet-0.01; Enet-0.5), Ridge regression (glmnet), the proposed method (glmnet-M) and the Lasso method.


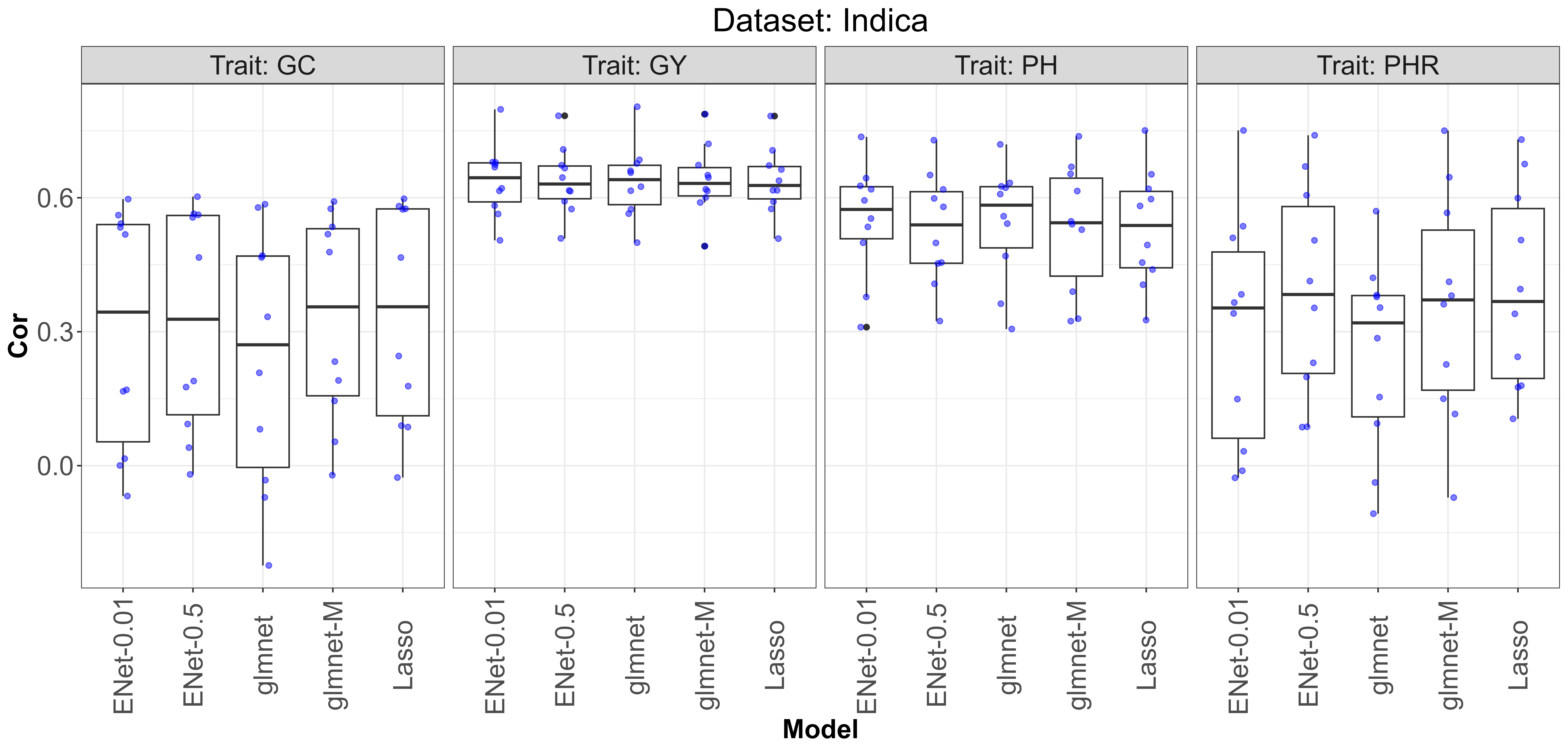


**Figure S11**. Box Plot prediction performance in terms of Pearson’s Correlation (Cor) between observed and predicted values through ten-fold cross-validation for each of the four traits (GC, GY, PH and PHR) of the **Indica** dataset with Elastic net with $\alpha=0.01 \mathrm{and} 0.5$(Enet-0.01; Enet-0.5), Ridge regression (glmnet), the proposed method (glmnet-M) and the Lasso method.


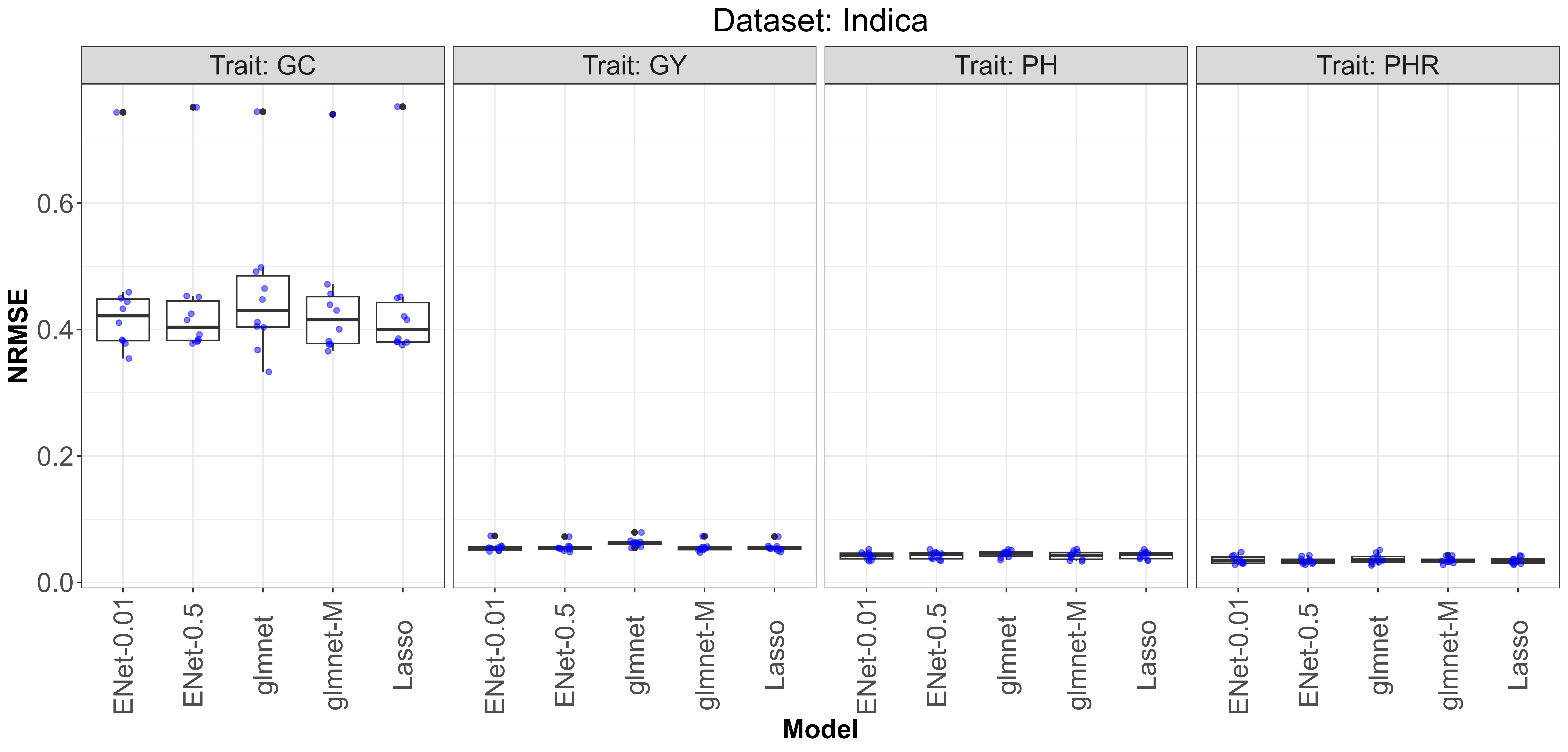


**Figure S*12***. Box Plot prediction performance in terms of the Normalized Root Mean Squared Error (NRMSE) between observed and predicted values through ten-fold cross-validation for each of the four traits (GC, GY, PH and PHR) of the **Indica** dataset with Elastic net with $\alpha=0.01 \mathrm{and} 0.5$(Enet-0.01; Enet-0.5), Ridge regression (glmnet), the proposed method (glmnet-M) and the Lasso method.


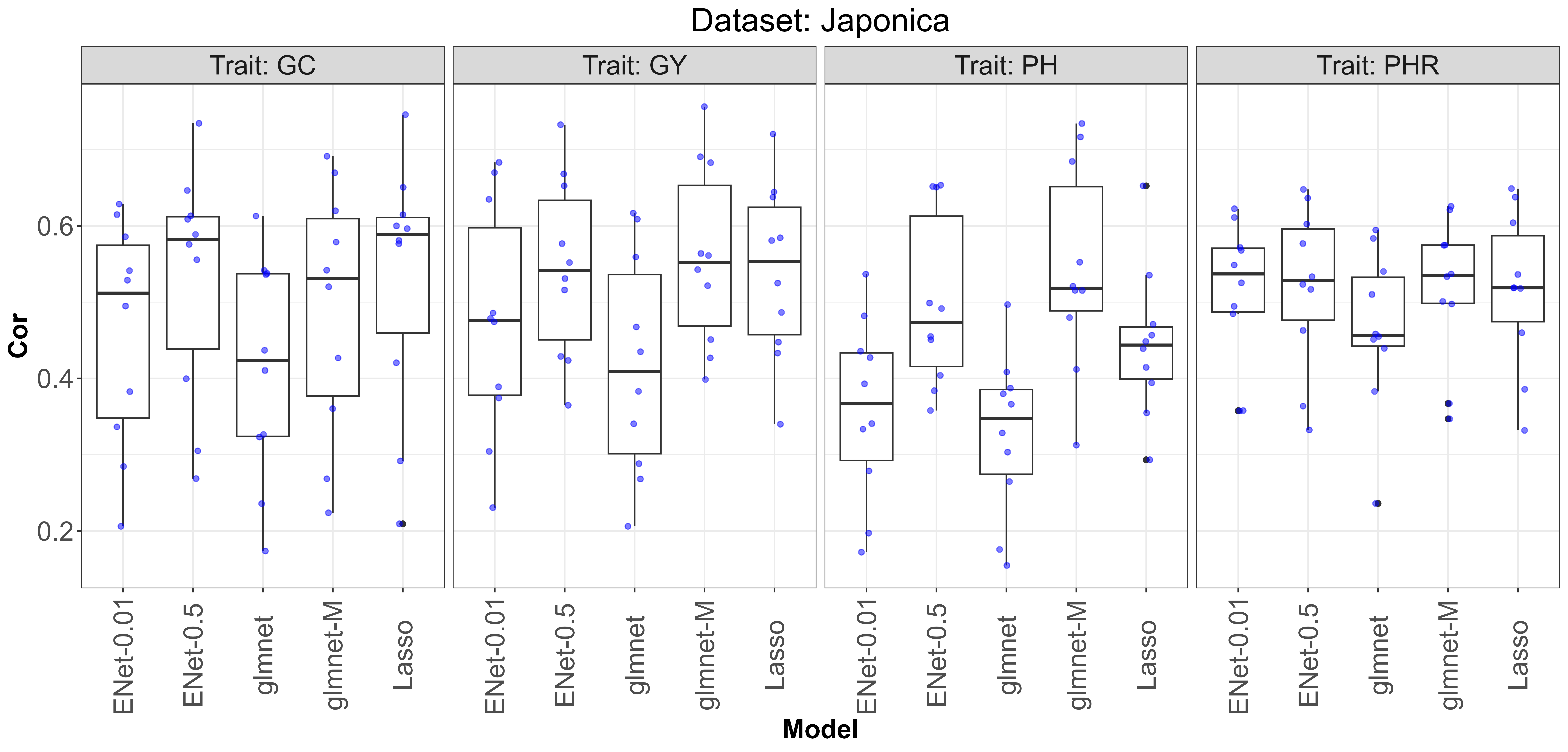


**Figure 13**. Box Plot prediction performance in terms of Pearson’s Correlation (Cor) between observed and predicted values through ten-fold cross-validation for each of the four traits (GC, GY, PH y PHR) of the **Japonica** dataset with Elastic net with $\alpha=0.01 \mathrm{and} 0.5$(Enet-0.01; Enet-0.5), Ridge regression (glmnet), the proposed method (glmnet-M) and the Lasso method.


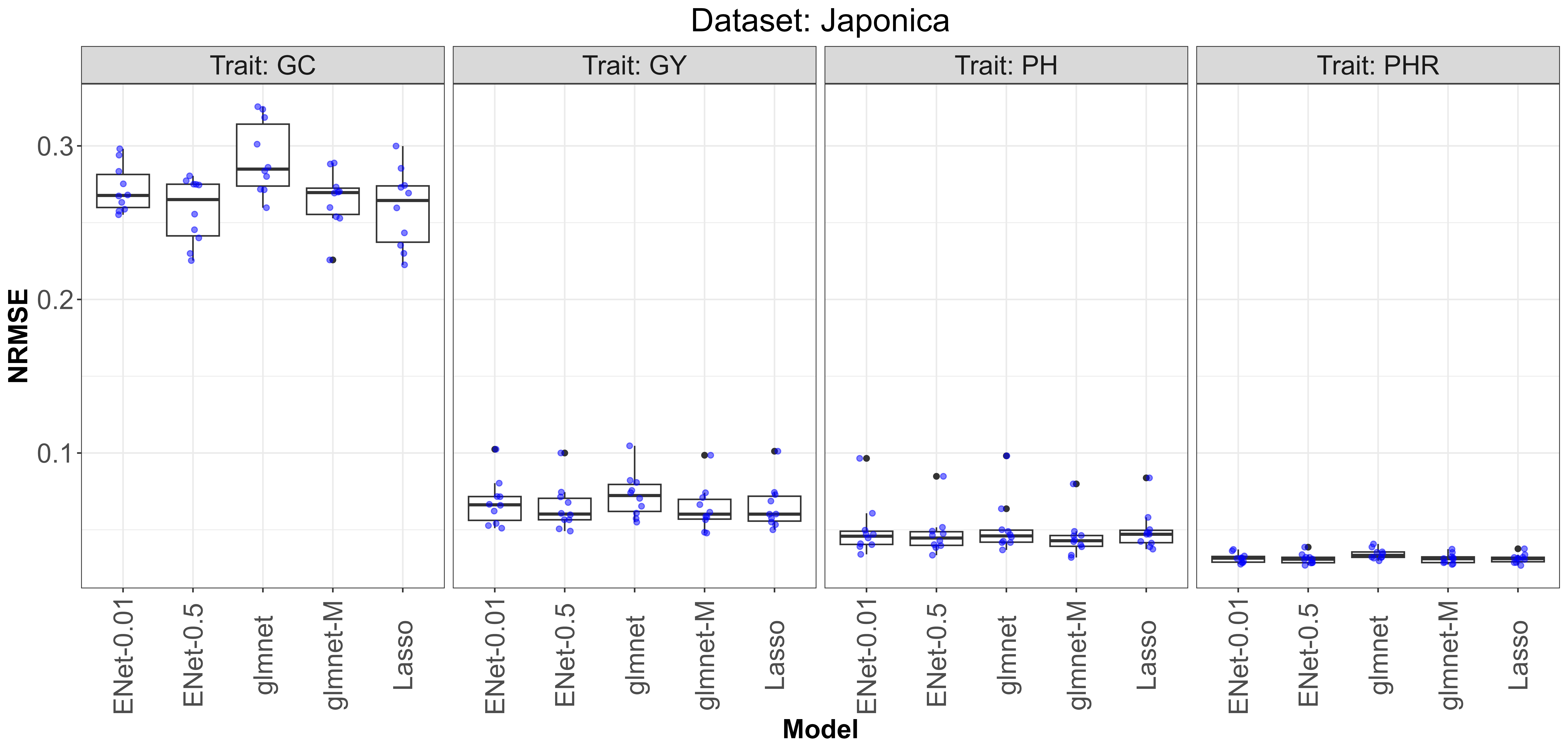


**Figure S*14***. Box Plot prediction performance in terms of the Normalized Root Mean Squared Error (NRMSE) between observed and predicted values through ten-fold cross-validation for each of the four traits (GC, GY, PH y PHR) of the **Japonica** dataset with Elastic net with $\alpha=0.01 \mathrm{and} 0.5$(Enet-0.01; Enet-0.5), Ridge regression (glmnet), the proposed method (glmnet-M) and the Lasso method.


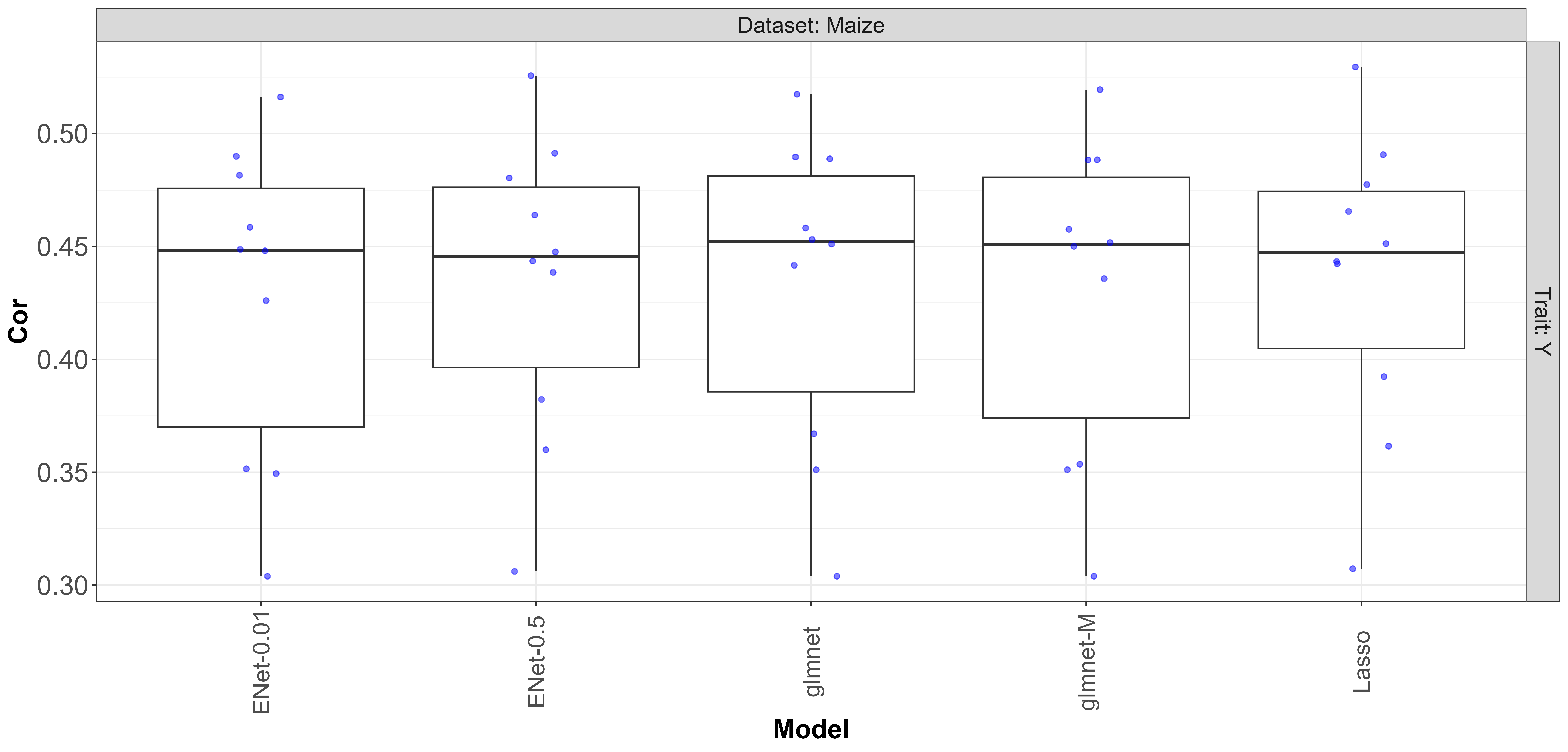


**Figure S15**. Box Plot prediction performance in terms of Pearson’s Correlation (Cor) between observed and predicted values through ten-fold cross-validation for the unique trait (Y) of the **Maize** dataset with Elastic net with $\alpha=0.01 \mathrm{and} 0.5$(Enet-0.01; Enet-0.5), Ridge regression (glmnet), the proposed method (glmnet-M) and the Lasso method.


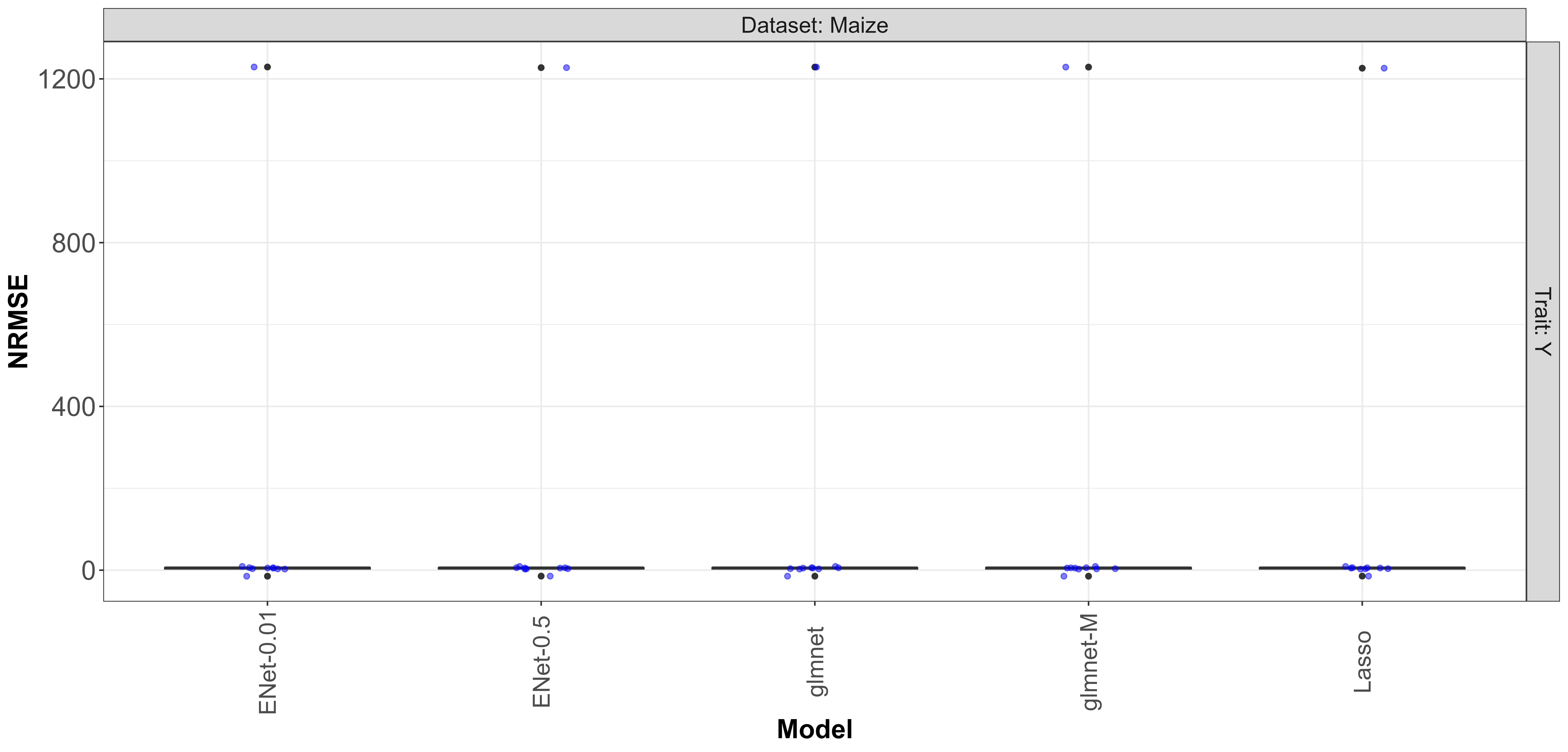


**Figure S*16***. Box plot prediction performance in terms of the Normalized Root Mean Squared Error (NRMSE) between observed and predicted values through ten-fold cross-validation for the unique trait (Y) of the **Maize** dataset with Elastic net with $\alpha=0.01 \mathrm{and} 0.5$(Enet-0.01; Enet-0.5), Ridge regression (glmnet), the proposed method (glmnet-M) and the Lasso method.


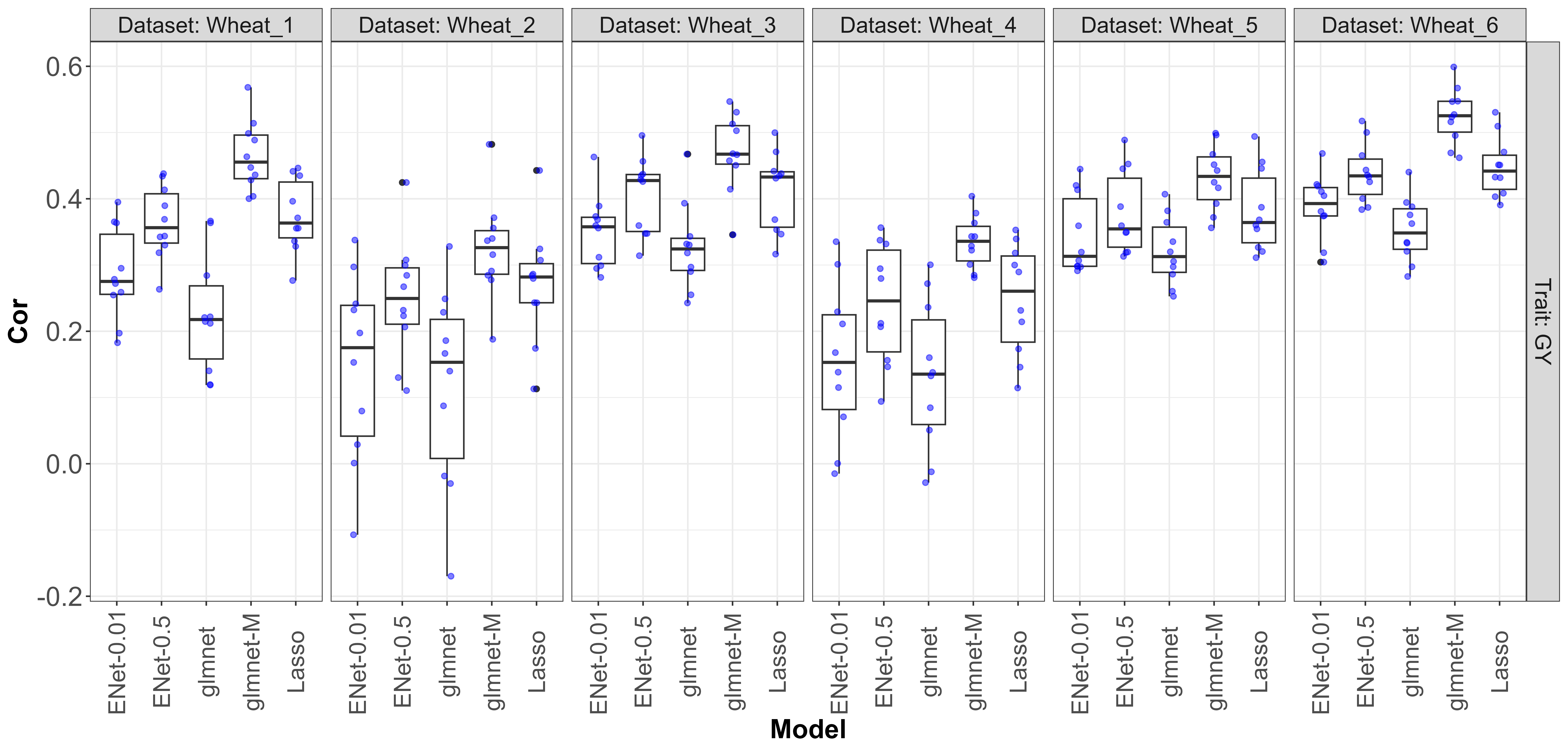


**Figure S17**. Box plot prediction performance in terms of Pearson’s correlation (Cor) between observed and predicted values through ten-fold cross-validation for the unique trait (GY) in each of the **Wheat_1-Wheat_6** datasets with Elastic net with $\alpha=0.01 \mathrm{and} 0.5$(Enet-0.01; Enet-0.5), Ridge regression (glmnet), the proposed method (glmnet-M) and the Lasso method.


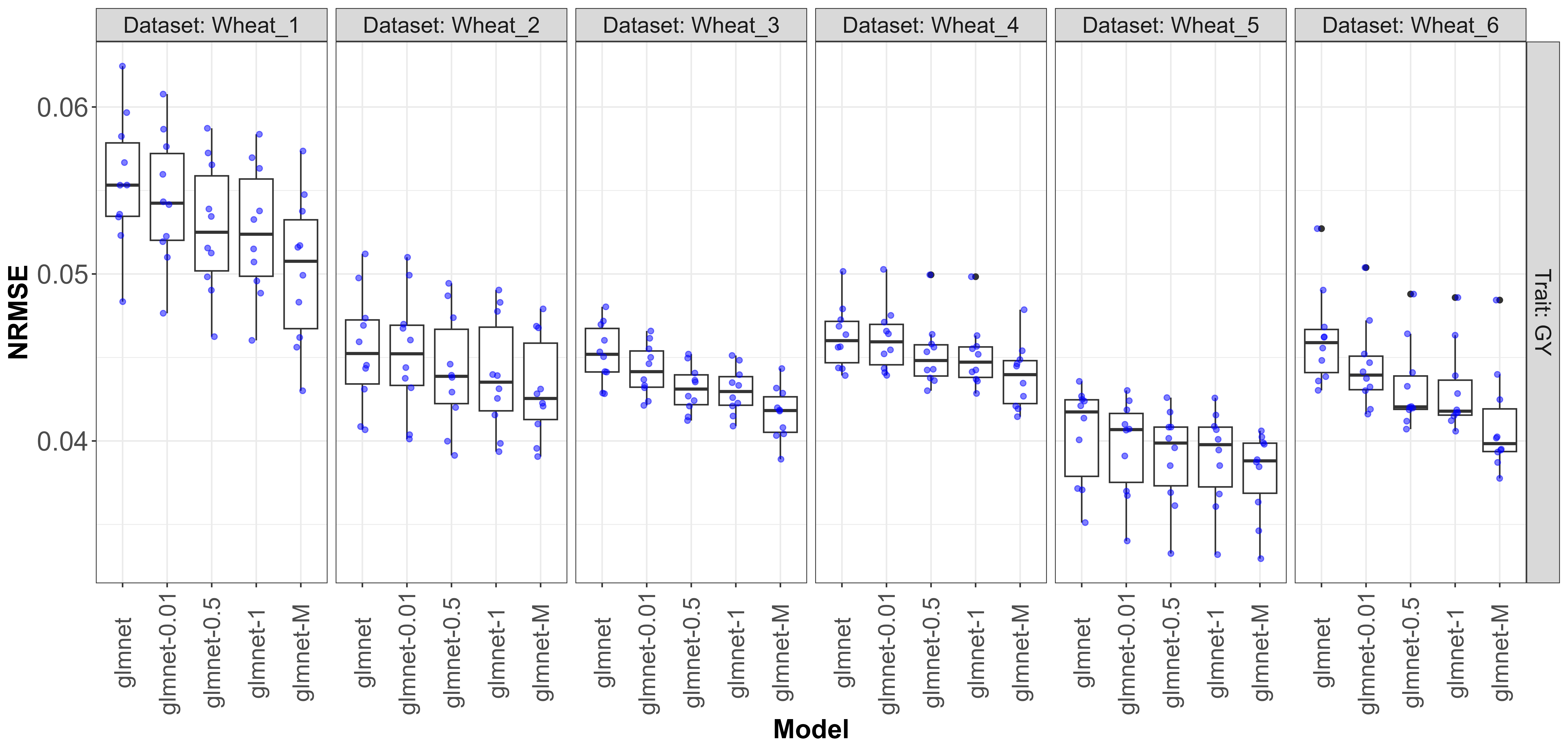


**Figure S*18***. Box Plot prediction performance in terms of the Normalized Root Mean Squared Error (NRMSE) between observed and predicted values through ten-fold cross-validation for the unique trait (GY) in each of the **Wheat_1-Wheat_6** datasets with Elastic net with $\alpha=0.01 \mathrm{and} 0.5$(Enet-0.01; Enet-0.5), Ridge regression (glmnet), the proposed method (glmnet-M) and the Lasso method.


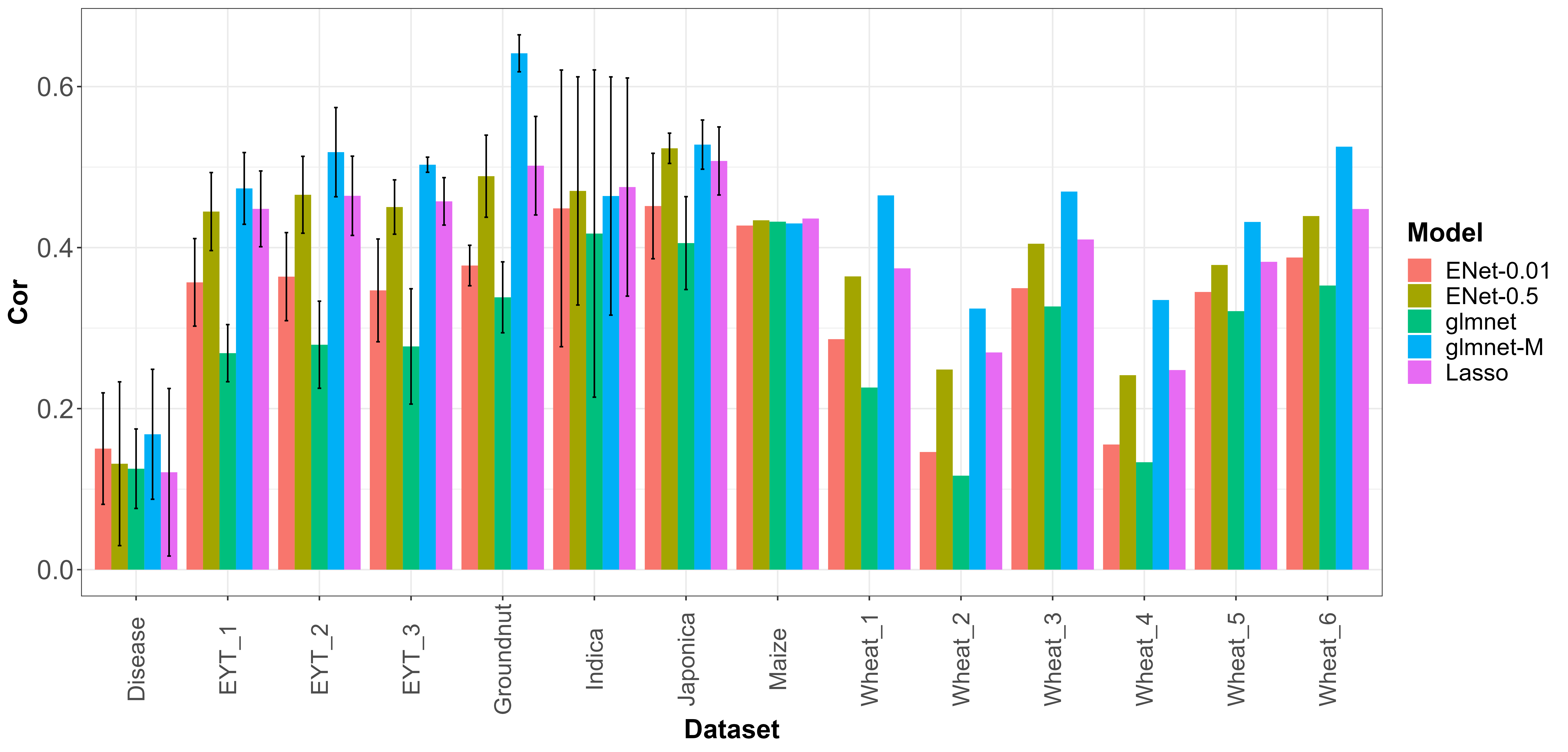


**Figure S*19***. Average Pearson’s Correlation (Cor) across traits with Elastic net with $\alpha=0.01 \mathrm{and} 0.5$(Enet-0.01; Enet-0.5), Ridge regression (glmnet), the proposed method (glmnet-M) and the Lasso method in each dataset (Dataset). The limits of the vertical lines in each bar indicate the average minus and plus one standard deviation (SD) of the correlation obtained across traits.


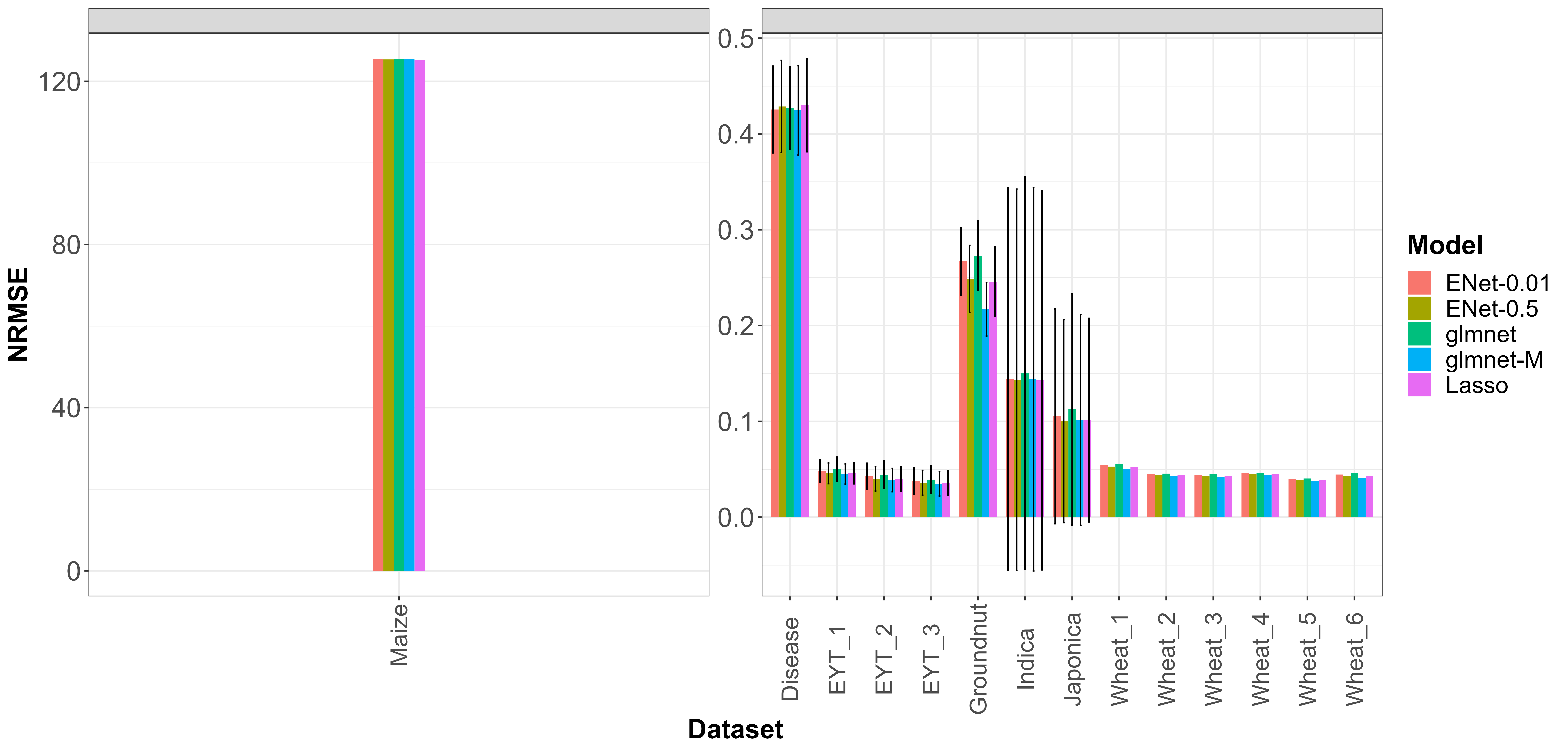


**Figure S*20***. Average Normalized Root Mean Squared Error (NRMSE) across traits with Elastic net with $\alpha=0.01 \mathrm{and} 0.5$(Enet-0.01; Enet-0.5), Ridge regression (glmnet), the proposed method (glmnet-M) and the Lasso method in each dataset (Dataset). The limits of the vertical lines in each bar indicate the average minus and plus one standard deviation (SD) of the NRMSE obtained across traits.

**Table S1** Average Normalized Root Mean Squared Error (NRMSE) and average Pearson’s Correlation (Cor) across ten-fold cross-validation, with Elastic net with $\alpha=0.01 \mathrm{and} 0.5$(Enet-0.01; Enet-0.5), Ridge regression (glmnet), the proposed method (glmnet-M) and the Lasso method in each dataset (Dataset) and for each trait (Trait). SD represents the standard deviation across folds.

| **Method** | **Dataset** | **Trait** | **NRMSE (SD)** | **Cor (SD)** |
| --- | --- | --- | --- | --- |
| Enet-0.01 | Disease | PTR | 0.4259 (0.039) | 0.1422 (0.1319) |
| Enet-0.5 | Disease | PTR | 0.4338 (0.0408) | 0.0921 (0.1005) |
| Lasso | Disease | PTR | 0.4361 (0.0406) | 0.0739 (0.1165) |
| glmnet | Disease | PTR | 0.4285 (0.041) | 0.0806 (0.1593) |
| glmnet-M | Disease | PTR | 0.425 (0.0389) | 0.1726 (0.1257) |
| Enet-0.01 | Disease | SB | 0.38 (0.031) | 0.2233 (0.1709) |
| Enet-0.5 | Disease | SB | 0.3782 (0.0317) | 0.247 (0.1388) |
| Lasso | Disease | SB | 0.3786 (0.0319) | 0.2402 (0.1406) |
| glmnet | Disease | SB | 0.3835 (0.0318) | 0.1783 (0.1652) |
| glmnet-M | Disease | SB | 0.3777 (0.0323) | 0.2464 (0.1416) |
| Enet-0.01 | Disease | SN | 0.4705 (0.0337) | 0.0855 (0.09) |
| Enet-0.5 | Disease | SN | 0.474 (0.0311) | 0.0554 (0.091) |
| Lasso | Disease | SN | 0.475 (0.0305) | 0.0487 (0.093) |
| glmnet | Disease | SN | 0.4694 (0.0343) | 0.1171 (0.1092) |
| glmnet-M | Disease | SN | 0.4711 (0.0318) | 0.0852 (0.0892) |
| Enet-0.01 | EYT | DTHD | 0.0594 (0.0049) | 0.4093 (0.0618) |
| Enet-0.5 | EYT | DTHD | 0.0556 (0.0054) | 0.5052 (0.0818) |
| Lasso | EYT | DTHD | 0.0554 (0.0056) | 0.5082 (0.0854) |
| glmnet | EYT | DTHD | 0.0624 (0.005) | 0.2985 (0.074) |
| glmnet-M | EYT | DTHD | 0.0549 (0.0055) | 0.521 (0.0758) |
| Enet-0.01 | EYT | DTMT | 0.0382 (0.0027) | 0.3687 (0.0754) |
| Enet-0.5 | EYT | DTMT | 0.0363 (0.0026) | 0.4577 (0.1012) |
| Lasso | EYT | DTMT | 0.0362 (0.0026) | 0.4606 (0.1037) |
| glmnet | EYT | DTMT | 0.0397 (0.003) | 0.2857 (0.0677) |
| glmnet-M | EYT | DTMT | 0.0355 (0.0028) | 0.4933 (0.0937) |
| Enet-0.01 | EYT | GY | 0.0569 (0.0039) | 0.3687 (0.137) |
| Enet-0.5 | EYT | GY | 0.0548 (0.0035) | 0.4242 (0.1035) |
| Lasso | EYT | GY | 0.0549 (0.0037) | 0.4225 (0.0998) |
| glmnet | EYT | GY | 0.0593 (0.0046) | 0.2732 (0.1524) |
| glmnet-M | EYT | GY | 0.0537 (0.0038) | 0.4625 (0.0929) |
| Enet-0.01 | EYT | Height | 0.0382 (0.0049) | 0.2804 (0.0878) |
| Enet-0.5 | EYT | Height | 0.0365 (0.0047) | 0.3918 (0.0719) |
| Lasso | EYT | Height | 0.0364 (0.0048) | 0.401 (0.0786) |
| glmnet | EYT | Height | 0.039 (0.005) | 0.2179 (0.0977) |
| glmnet-M | EYT | Height | 0.0362 (0.0046) | 0.417 (0.0949) |
| Enet-0.01 | EYT | DTHD | 0.048 (0.005) | 0.2894 (0.0805) |
| Enet-0.5 | EYT | DTHD | 0.0458 (0.0036) | 0.4032 (0.106) |
| Lasso | EYT | DTHD | 0.0457 (0.0034) | 0.4034 (0.1115) |
| glmnet | EYT | DTHD | 0.0491 (0.0053) | 0.2141 (0.0692) |
| glmnet-M | EYT | DTHD | 0.0445 (0.0037) | 0.4644 (0.0966) |
| Enet-0.01 | EYT | DTMT | 0.0278 (0.0029) | 0.3646 (0.0812) |
| Enet-0.5 | EYT | DTMT | 0.026 (0.0023) | 0.4839 (0.076) |
| Lasso | EYT | DTMT | 0.026 (0.0023) | 0.4873 (0.0787) |
| glmnet | EYT | DTMT | 0.0289 (0.0029) | 0.2905 (0.0845) |
| glmnet-M | EYT | DTMT | 0.0248 (0.0023) | 0.5537 (0.0746) |
| Enet-0.01 | EYT | GY | 0.0588 (0.0044) | 0.4195 (0.0583) |
| Enet-0.5 | EYT | GY | 0.055 (0.0042) | 0.5162 (0.0521) |
| Lasso | EYT | GY | 0.0549 (0.0041) | 0.5172 (0.0508) |
| glmnet | EYT | GY | 0.0616 (0.0046) | 0.3447 (0.0527) |
| glmnet-M | EYT | GY | 0.0523 (0.0037) | 0.5773 (0.0706) |
| Enet-0.01 | EYT | Height | 0.0356 (0.0033) | 0.3821 (0.0813) |
| Enet-0.5 | EYT | Height | 0.0337 (0.0022) | 0.4589 (0.1037) |
| Lasso | EYT | Height | 0.0339 (0.0022) | 0.4492 (0.1045) |
| glmnet | EYT | Height | 0.0373 (0.0037) | 0.2679 (0.1167) |
| glmnet-M | EYT | Height | 0.0333 (0.0024) | 0.4787 (0.1014) |
| Enet-0.01 | EYT | DTHD | 0.0388 (0.0016) | 0.3015 (0.1081) |
| Enet-0.5 | EYT | DTHD | 0.0359 (0.0024) | 0.4672 (0.0812) |
| Lasso | EYT | DTHD | 0.0357 (0.0023) | 0.4804 (0.0722) |
| glmnet | EYT | DTHD | 0.0398 (0.0015) | 0.2176 (0.1001) |
| glmnet-M | EYT | DTHD | 0.0353 (0.0026) | 0.4918 (0.0859) |
| Enet-0.01 | EYT | DTMT | 0.0225 (0.0013) | 0.29 (0.0645) |
| Enet-0.5 | EYT | DTMT | 0.0215 (0.0015) | 0.4004 (0.0762) |
| Lasso | EYT | DTMT | 0.0214 (0.0015) | 0.4142 (0.0736) |
| glmnet | EYT | DTMT | 0.023 (0.0013) | 0.2281 (0.0559) |
| glmnet-M | EYT | DTMT | 0.0204 (0.0014) | 0.499 (0.0751) |
| Enet-0.01 | EYT | GY | 0.0557 (0.0031) | 0.3683 (0.067) |
| Enet-0.5 | EYT | GY | 0.0529 (0.0026) | 0.4604 (0.0539) |
| Lasso | EYT | GY | 0.0528 (0.0026) | 0.4661 (0.055) |
| glmnet | EYT | GY | 0.0579 (0.0032) | 0.2896 (0.0797) |
| glmnet-M | EYT | GY | 0.0514 (0.0028) | 0.5081 (0.044) |
| Enet-0.01 | EYT | Height | 0.0339 (0.0022) | 0.4274 (0.0681) |
| Enet-0.5 | EYT | Height | 0.0328 (0.0019) | 0.4734 (0.0493) |
| Lasso | EYT | Height | 0.0329 (0.0019) | 0.4689 (0.0478) |
| glmnet | EYT | Height | 0.0357 (0.0024) | 0.3735 (0.0944) |
| glmnet-M | EYT | Height | 0.0319 (0.0017) | 0.5128 (0.0444) |
| Enet-0.01 | Groundnut | NPP | 0.2556 (0.0299) | 0.368 (0.2176) |
| Enet-0.5 | Groundnut | NPP | 0.2253 (0.0251) | 0.5535 (0.1137) |
| Lasso | Groundnut | NPP | 0.2184 (0.0249) | 0.5863 (0.1038) |
| glmnet | Groundnut | NPP | 0.2633 (0.0309) | 0.2823 (0.2266) |
| glmnet-M | Groundnut | NPP | 0.2022 (0.0246) | 0.6679 (0.0856) |
| Enet-0.01 | Groundnut | PYPP | 0.235 (0.0378) | 0.376 (0.2075) |
| Enet-0.5 | Groundnut | PYPP | 0.2225 (0.0382) | 0.4637 (0.1762) |
| Lasso | Groundnut | PYPP | 0.2216 (0.0387) | 0.4666 (0.1744) |
| glmnet | Groundnut | PYPP | 0.2395 (0.0381) | 0.348 (0.2086) |
| glmnet-M | Groundnut | PYPP | 0.1931 (0.0337) | 0.6334 (0.1233) |
| Enet-0.01 | Groundnut | SYPP | 0.2608 (0.0387) | 0.354 (0.1897) |
| Enet-0.5 | Groundnut | SYPP | 0.2486 (0.0364) | 0.4357 (0.1509) |
| Lasso | Groundnut | SYPP | 0.2461 (0.0357) | 0.4485 (0.1474) |
| glmnet | Groundnut | SYPP | 0.2645 (0.0397) | 0.3335 (0.1941) |
| glmnet-M | Groundnut | SYPP | 0.2167 (0.0299) | 0.6141 (0.1103) |
| Enet-0.01 | Groundnut | YPH | 0.3173 (0.0522) | 0.4129 (0.2358) |
| Enet-0.5 | Groundnut | YPH | 0.2981 (0.0541) | 0.5017 (0.2241) |
| Lasso | Groundnut | YPH | 0.2967 (0.0548) | 0.5054 (0.2219) |
| glmnet | Groundnut | YPH | 0.3245 (0.0522) | 0.389 (0.2312) |
| glmnet-M | Groundnut | YPH | 0.2562 (0.0418) | 0.6497 (0.1553) |
| Enet-0.01 | Indica | GC | 0.4437 (0.1111) | 0.3036 (0.2703) |
| Enet-0.5 | Indica | GC | 0.4415 (0.1126) | 0.323 (0.2488) |
| Lasso | Indica | GC | 0.4393 (0.1139) | 0.3367 (0.2465) |
| glmnet | Indica | GC | 0.4568 (0.1139) | 0.2396 (0.2903) |
| glmnet-M | Indica | GC | 0.4439 (0.1105) | 0.3299 (0.2335) |
| Enet-0.01 | Indica | GY | 0.0556 (0.0069) | 0.6387 (0.0806) |
| Enet-0.5 | Indica | GY | 0.0552 (0.0066) | 0.6382 (0.0759) |
| Lasso | Indica | GY | 0.0552 (0.0066) | 0.637 (0.0755) |
| glmnet | Indica | GY | 0.063 (0.0065) | 0.636 (0.0825) |
| glmnet-M | Indica | GY | 0.0549 (0.0069) | 0.6392 (0.0793) |
| Enet-0.01 | Indica | PH | 0.0421 (0.006) | 0.5495 (0.1274) |
| Enet-0.5 | Indica | PH | 0.0425 (0.006) | 0.5314 (0.1243) |
| Lasso | Indica | PH | 0.0426 (0.006) | 0.5321 (0.1296) |
| glmnet | Indica | PH | 0.0451 (0.0054) | 0.5447 (0.1294) |
| glmnet-M | Indica | PH | 0.0425 (0.0068) | 0.5334 (0.1444) |
| Enet-0.01 | Indica | PHR | 0.0358 (0.0066) | 0.3029 (0.2613) |
| Enet-0.5 | Indica | PHR | 0.0341 (0.0051) | 0.3889 (0.2376) |
| Lasso | Indica | PHR | 0.0342 (0.0051) | 0.3949 (0.2235) |
| glmnet | Indica | PHR | 0.0369 (0.0078) | 0.2493 (0.2163) |
| glmnet-M | Indica | PHR | 0.0349 (0.0048) | 0.3537 (0.2553) |
| Enet-0.01 | Japonica | GC | 0.272 (0.0152) | 0.4603 (0.148) |
| Enet-0.5 | Japonica | GC | 0.2578 (0.0212) | 0.5295 (0.1529) |
| Lasso | Japonica | GC | 0.2592 (0.0255) | 0.5286 (0.1679) |
| glmnet | Japonica | GC | 0.2921 (0.0236) | 0.4134 (0.146) |
| glmnet-M | Japonica | GC | 0.2652 (0.0185) | 0.49 (0.1639) |
| Enet-0.01 | Japonica | GY | 0.0678 (0.0153) | 0.4724 (0.1539) |
| Enet-0.5 | Japonica | GY | 0.0647 (0.0149) | 0.5445 (0.1176) |
| Lasso | Japonica | GY | 0.0653 (0.015) | 0.5399 (0.1152) |
| glmnet | Japonica | GY | 0.0726 (0.0146) | 0.4172 (0.1452) |
| glmnet-M | Japonica | GY | 0.0641 (0.0148) | 0.5594 (0.1192) |
| Enet-0.01 | Japonica | PH | 0.05 (0.0178) | 0.3596 (0.1187) |
| Enet-0.5 | Japonica | PH | 0.0474 (0.0142) | 0.4997 (0.1138) |
| Lasso | Japonica | PH | 0.0494 (0.0134) | 0.4459 (0.0981) |
| glmnet | Japonica | PH | 0.0515 (0.0178) | 0.3265 (0.1055) |
| glmnet-M | Japonica | PH | 0.0451 (0.0133) | 0.5443 (0.1345) |
| Enet-0.01 | Japonica | PHR | 0.0315 (0.0032) | 0.5141 (0.0936) |
| Enet-0.5 | Japonica | PHR | 0.031 (0.0034) | 0.5195 (0.1069) |
| Lasso | Japonica | PHR | 0.0312 (0.003) | 0.5159 (0.1025) |
| glmnet | Japonica | PHR | 0.0342 (0.0034) | 0.465 (0.1046) |
| glmnet-M | Japonica | PHR | 0.0312 (0.0032) | 0.5178 (0.0954) |
| Enet-0.01 | Maize | Y | 125.5246 (387.7967) | 0.4274 (0.0696) |
| Enet-0.5 | Maize | Y | 125.3677 (387.2929) | 0.4339 (0.0662) |
| Lasso | Maize | Y | 125.234 (386.8583) | 0.4361 (0.0655) |
| glmnet | Maize | Y | 125.4932 (387.721) | 0.4322 (0.0687) |
| glmnet-M | Maize | Y | 125.49 (387.724) | 0.43 (0.0702) |
| Enet-0.01 | Wheat | GY | 0.0544 (0.0039) | 0.2862 (0.0705) |
| Enet-0.5 | Wheat | GY | 0.0527 (0.0039) | 0.3642 (0.0555) |
| Lasso | Wheat | GY | 0.0525 (0.0039) | 0.3742 (0.0554) |
| glmnet | Wheat | GY | 0.0555 (0.0039) | 0.2262 (0.0898) |
| glmnet-M | Wheat | GY | 0.0502 (0.0044) | 0.4648 (0.0529) |
| Enet-0.01 | Wheat | GY | 0.0452 (0.0036) | 0.1461 (0.1422) |
| Enet-0.5 | Wheat | GY | 0.0441 (0.0034) | 0.2485 (0.0909) |
| Lasso | Wheat | GY | 0.0439 (0.0034) | 0.2697 (0.0882) |
| glmnet | Wheat | GY | 0.0454 (0.0034) | 0.1167 (0.1509) |
| glmnet-M | Wheat | GY | 0.0431 (0.003) | 0.3242 (0.076) |
| Enet-0.01 | Wheat | GY | 0.0442 (0.0015) | 0.3496 (0.0549) |
| Enet-0.5 | Wheat | GY | 0.0431 (0.0013) | 0.4048 (0.0584) |
| Lasso | Wheat | GY | 0.043 (0.0013) | 0.41 (0.0599) |
| glmnet | Wheat | GY | 0.0452 (0.0017) | 0.3268 (0.0658) |
| glmnet-M | Wheat | GY | 0.0416 (0.0015) | 0.4696 (0.0592) |
| Enet-0.01 | Wheat | GY | 0.046 (0.0019) | 0.1554 (0.1175) |
| Enet-0.5 | Wheat | GY | 0.0452 (0.0019) | 0.2415 (0.0912) |
| Lasso | Wheat | GY | 0.0451 (0.0019) | 0.2479 (0.0843) |
| glmnet | Wheat | GY | 0.0462 (0.0019) | 0.1334 (0.113) |
| glmnet-M | Wheat | GY | 0.0438 (0.0019) | 0.3349 (0.0399) |
| Enet-0.01 | Wheat | GY | 0.0396 (0.0028) | 0.3449 (0.0597) |
| Enet-0.5 | Wheat | GY | 0.039 (0.0028) | 0.3784 (0.0628) |
| Lasso | Wheat | GY | 0.0389 (0.0028) | 0.3823 (0.0626) |
| glmnet | Wheat | GY | 0.0404 (0.0029) | 0.321 (0.0511) |
| glmnet-M | Wheat | GY | 0.038 (0.0025) | 0.4318 (0.0489) |
| Enet-0.01 | Wheat | GY | 0.0445 (0.0026) | 0.3877 (0.0489) |
| Enet-0.5 | Wheat | GY | 0.0432 (0.0025) | 0.4391 (0.0448) |
| Lasso | Wheat | GY | 0.043 (0.0025) | 0.4479 (0.0453) |
| glmnet | Wheat | GY | 0.0461 (0.0028) | 0.3528 (0.0482) |
| glmnet-M | Wheat | GY | 0.041 (0.0031) | 0.5253 (0.0424) |

**Table S2** Average Normalized Root Mean Squared Error (NRMSE) and average Pearson’s Correlation (Cor) across traits in each dataset (Dataset) with Elastic net with $\alpha=0.01 \mathrm{and} 0.5$(Enet-0.01; Enet-0.5), Ridge regression (glmnet), the proposed method (glmnet-M) and the Lasso method. SD represents the standard deviation across traits of the mean values obtained across folds. Note that because there is only one trait in the Maize and Wheat_1-Wheat_6 datasets, the corresponding standard deviation is 0.

| **Method** | **Dataset** | **NRMSE (SD)** | **Cor (SD)** |
| --- | --- | --- | --- |
| ENet-0.01 | Disease | 0.4255 (0.0452) | 0.1503 (0.0692) |
| ENet-0.5 | Disease | 0.4286 (0.0481) | 0.1315 (0.1017) |
| Lasso | Disease | 0.4299 (0.0484) | 0.1209 (0.104) |
| glmnet | Disease | 0.4271 (0.0429) | 0.1253 (0.0493) |
| glmnet-M | Disease | 0.4246 (0.0466) | 0.1681 (0.0806) |
| ENet-0.01 | EYT_1 | 0.0482 (0.0115) | 0.3568 (0.0543) |
| ENet-0.5 | EYT_1 | 0.0458 (0.0108) | 0.4447 (0.0484) |
| Lasso | EYT_1 | 0.0457 (0.0108) | 0.4481 (0.047) |
| glmnet | EYT_1 | 0.0501 (0.0124) | 0.2688 (0.0354) |
| glmnet-M | EYT_1 | 0.045 (0.0106) | 0.4735 (0.0445) |
| ENet-0.01 | EYT_2 | 0.0426 (0.0136) | 0.3639 (0.0547) |
| ENet-0.5 | EYT_2 | 0.0401 (0.0128) | 0.4656 (0.0477) |
| Lasso | EYT_2 | 0.0401 (0.0127) | 0.4643 (0.0492) |
| glmnet | EYT_2 | 0.0442 (0.0142) | 0.2793 (0.054) |
| glmnet-M | EYT_2 | 0.0387 (0.0121) | 0.5185 (0.0554) |
| ENet-0.01 | EYT_3 | 0.0377 (0.0137) | 0.3468 (0.0638) |
| ENet-0.5 | EYT_3 | 0.0358 (0.0129) | 0.4503 (0.0337) |
| Lasso | EYT_3 | 0.0357 (0.0129) | 0.4574 (0.0294) |
| glmnet | EYT_3 | 0.0391 (0.0144) | 0.2772 (0.0716) |
| glmnet-M | EYT_3 | 0.0348 (0.0128) | 0.5029 (0.0093) |
| ENet-0.01 | Groundnut | 0.2672 (0.0351) | 0.3777 (0.0251) |
| ENet-0.5 | Groundnut | 0.2486 (0.0349) | 0.4887 (0.0509) |
| Lasso | Groundnut | 0.2457 (0.0361) | 0.5017 (0.0612) |
| glmnet | Groundnut | 0.2729 (0.0362) | 0.3382 (0.044) |
| glmnet-M | Groundnut | 0.217 (0.0278) | 0.6413 (0.0229) |
| ENet-0.01 | Indica | 0.1443 (0.1997) | 0.4487 (0.1718) |
| ENet-0.5 | Indica | 0.1433 (0.1989) | 0.4704 (0.1417) |
| Lasso | Indica | 0.1428 (0.1978) | 0.4752 (0.1354) |
| glmnet | Indica | 0.1505 (0.2045) | 0.4174 (0.2032) |
| glmnet-M | Indica | 0.1441 (0.2) | 0.464 (0.1479) |
| ENet-0.01 | Japonica | 0.1053 (0.1121) | 0.4516 (0.0654) |
| ENet-0.5 | Japonica | 0.1002 (0.1059) | 0.5233 (0.0188) |
| Lasso | Japonica | 0.1013 (0.1061) | 0.5076 (0.0422) |
| glmnet | Japonica | 0.1126 (0.1206) | 0.4056 (0.0576) |
| glmnet-M | Japonica | 0.1014 (0.11) | 0.5279 (0.0305) |
| ENet-0.01 | Maize | 125.5246 (0) | 0.4274 (0) |
| ENet-0.5 | Maize | 125.3677 (0) | 0.4339 (0) |
| Lasso | Maize | 125.234 (0) | 0.4361 (0) |
| glmnet | Maize | 125.4932 (0) | 0.4322 (0) |
| glmnet-M | Maize | 125.49 (0) | 0.43 (0) |
| ENet-0.01 | Wheat_1 | 0.0544 (0) | 0.2862 (0) |
| ENet-0.5 | Wheat_1 | 0.0527 (0) | 0.3642 (0) |
| Lasso | Wheat_1 | 0.0525 (0) | 0.3742 (0) |
| glmnet | Wheat_1 | 0.0555 (0) | 0.2262 (0) |
| glmnet-M | Wheat_1 | 0.0502 (0) | 0.4648 (0) |
| ENet-0.01 | Wheat_2 | 0.0452 (0) | 0.1461 (0) |
| ENet-0.5 | Wheat_2 | 0.0441 (0) | 0.2485 (0) |
| Lasso | Wheat_2 | 0.0439 (0) | 0.2697 (0) |
| glmnet | Wheat_2 | 0.0454 (0) | 0.1167 (0) |
| glmnet-M | Wheat_2 | 0.0431 (0) | 0.3242 (0) |
| ENet-0.01 | Wheat_3 | 0.0442 (0) | 0.3496 (0) |
| ENet-0.5 | Wheat_3 | 0.0431 (0) | 0.4048 (0) |
| Lasso | Wheat_3 | 0.043 (0) | 0.41 (0) |
| glmnet | Wheat_3 | 0.0452 (0) | 0.3268 (0) |
| glmnet-M | Wheat_3 | 0.0416 (0) | 0.4696 (0) |
| ENet-0.01 | Wheat_4 | 0.046 (0) | 0.1554 (0) |
| ENet-0.5 | Wheat_4 | 0.0452 (0) | 0.2415 (0) |
| Lasso | Wheat_4 | 0.0451 (0) | 0.2479 (0) |
| glmnet | Wheat_4 | 0.0462 (0) | 0.1334 (0) |
| glmnet-M | Wheat_4 | 0.0438 (0) | 0.3349 (0) |
| ENet-0.01 | Wheat_5 | 0.0396 (0) | 0.3449 (0) |
| ENet-0.5 | Wheat_5 | 0.039 (0) | 0.3784 (0) |
| Lasso | Wheat_5 | 0.0389 (0) | 0.3823 (0) |
| glmnet | Wheat_5 | 0.0404 (0) | 0.321 (0) |
| glmnet-M | Wheat_5 | 0.038 (0) | 0.4318 (0) |
| ENet-0.01 | Wheat_6 | 0.0445 (0) | 0.3877 (0) |
| ENet-0.5 | Wheat_6 | 0.0432 (0) | 0.4391 (0) |
| Lasso | Wheat_6 | 0.043 (0) | 0.4479 (0) |
| glmnet | Wheat_6 | 0.0461 (0) | 0.3528 (0) |
| glmnet-M | Wheat_6 | 0.041 (0) | 0.5253 (0) |

**Table S3** Average Normalized Root Mean Squared Error (NRMSE) and average Pearson’s Correlation (Cor) across different datasets (Dataset) of the mean values obtained across traits, with Elastic net with $\alpha=0.01 \mathrm{and} 0.5$(Enet-0.01; Enet-0.5), Ridge regression (glmnet), the proposed method (glmnet-M) and the Lasso method. SD represents the standard deviation of the metric across different datasets of the mean values obtained across traits.

| **Model** | **NRMSE (SD)** | **Cor (SD)** |
| --- | --- | --- |
| ENet-0.01 | 9.0621 (33.5204) | 0.3281 (0.1056) |
| ENet-0.5 | 9.0484 (33.4791) | 0.3918 (0.1111) |
| Lasso | 9.0387 (33.4434) | 0.3959 (0.1108) |
| glmnet | 9.062 (33.5113) | 0.2872 (0.1055) |
| glmnet-M | 9.0538 (33.5128) | 0.4483 (0.1131) |
